# Supplementary material for: Prevalence and determinants of hyperglycaemia among adults in Bangladesh: results from a population-based national survey
Source: BMJ Open. 2019 Jul 24;9(7):e029674. doi: 10.1136/bmjopen-2019-029674 (PMC6661587; doi:10.1136/bmjopen-2019-029674)
Supplement: Author's manuscript [file bmjopen-2019-029674.draft_revisions.pdf]

BMJ Open

BMJ Open is committed to open peer review. As part of this commitment we make the peer review history of every article we publish publicly available.

When an article is published we post the peer reviewers' comments and the authors' responses online. We also post the versions of the paper that were used during peer review. These are the versions that the peer review comments apply to.

The versions of the paper that follow are the versions that were submitted during the peer review process. They are not the versions of record or the final published versions. They should not be cited or distributed as the published version of this manuscript.

BMJ Open is an open access journal and the full, final, typeset and author-corrected version of record of the manuscript is available on our site with no access controls, subscription charges or pay-per-view fees (http://bmjopen.bmj.com).

If you have any questions on BMJ Open's open peer review process please email info.bmjopen@bmj.com

BMJ Open

Prevalence and determinants of raised blood glucose among adults in Bangladesh: results from a population-based national survey

Journal:	*BMJ Open*
Manuscript ID	bmjopen-2019-029674
Article Type:	Research
Date Submitted by the Author:	05-Feb-2019
Complete List of Authors:	Islam, Jessica; World Health Organization Country Office for Bangladesh, ; University of North Carolina at Chapel Hill Gillings School of Global Public Health, Department of Epidemiology<br>Zaman, MM; World Health Organization, Dhaka, Bangladesh,<br>Bhuiyan, Mahfuz; World Health Organization Country Office for Bangladesh<br>Haq, Syed; Bangabandhu Sheikh Mujib Medical University, Rheumatology<br>Ahmed, Shamim; Bangabandhu Sheikh Mujib Medical University<br>Al-Qadir, Zahid; Bangabandhu Sheikh Mujib Medical University
Keywords:	non-communicable diseases, chronic disease, raised blood glucose, Bangladesh

SCHOLARONE™
Manuscripts

Prevalence and determinants of raised blood glucose among adults in Bangladesh: results from a population-based national survey

Jessica Y. Islam[1,2], M. Mostafa Zaman*[2], Mahfuzur Rahman Bhuiyan[2], Syed Atiqul Haq[3], Shamim Ahmed[3], Zahid- Al- Quadir[3]

[1]Department of Epidemiology, UNC Gillings School of Global Public Health, Chapel Hill, NC; Email: islamjy@email.unc.edu

[2]World Health Organization, Dhaka, Bangladesh; Email: zamanm@who.int; Email: mahfuzdoc@gmail.com

[3]Department of Rheumatology, Bangabandhu Sheikh Mujib Medical University, Shahbag, Dhaka, Bangladesh. Email: haqsyedatiqul@gmail.com; Email: dr_shamim_ahmed@yahoo.com; Email: drzahid1981@yahoo.com

*Corresponding Author:

Email : zamanm@who.int (MMZ)

For peer review only - http://bmjopen.bmj.com/site/about/guidelines.xhtml

Abstract

Objectives: With the increasing burden of non-communicable diseases in low- and middle-income countries, biological risk factors such as raised blood glucose are a major public health concern in Bangladesh. Nationally representative data of raised blood glucose prevalence starting from age ≥18 years are currently unavailable for Bangladeshi adults. The objective of this study was to assess the prevalence and determinants of raised blood glucose among adults in Bangladesh aged ≥18 years.

Study Design: Cross-sectional, population-based study

Setting and Participants: Data for this analysis were collected from a population-based nationally representative sample of 1843 adults, aged ≥18 years, from both urban and rural areas of Bangladesh. Demographic information, capillary blood glucose, blood pressure, height, weight, waist circumference, and treatment history were recorded.

Primary Outcome Measures: Raised blood glucose was defined as a random capillary blood glucose level of ≥11.1mmol/L or currently taking medication to control diabetes, based on self-report.

Results: Overall, the prevalence of raised blood glucose was 5.5% (95% CI: 4.5-6.6) and was significantly higher among urban (9.8%, 95%CI: 7.7-12.2) than rural residents (2.8%, 95% CI: 1.9 – 3.9). The age-standardized prevalence of raised blood glucose was 5.6% (95% CI: 4.6 – 6.8). Among both urban and rural residents, associated determinants of raised blood glucose included hypertension, and abdominal obesity. About 5% of the total population self-reported to have been previously diagnosed with diabetes; among these adults, over 25% were not taking medications to control their diabetes.

For peer review only

Conclusions: Our study found that about 1 out of 20 Bangladeshi adults aged ≥18 years have raised blood glucose. To control and prevent the development of diabetes, data from this study can be used to inform public health programming and provide descriptive information on surveillance of progress towards controlling diabetes in Bangladesh.

Keywords: non-communicable diseases, Bangladesh, raised blood glucose, diabetes, chronic disease

For peer review only

For peer review only - http://bmjopen.bmj.com/site/about/guidelines.xhtml

Article Summary

Strengths and Limitations of the Study

- This study utilized a multistage, geographically clustered, probability-based sampling approach to produce nationally representative data for Bangladesh.

- We included Bangladeshi adults aged 18 to 29 years to obtain novel data on the prevalence of diabetes and relevant non-communicable disease risk factors starting from this age group.

- We were able to estimate the prevalence of raised blood glucose using capillary blood glucose measured at random. However, we were unable to measure the prevalence of prediabetes and diabetes as we did not obtain blood sugar levels using standardized methods, such as fasting blood glucose or 2-hour post-prandial measurements.

- Due to the cross-sectional nature of the study design, we were unable to assess temporality of risk factors identified and our outcomes of interest.

- We assessed diabetes medication history based on self-report, however, we were unable to obtain medical records or prescription records of participants to confirm the self-reported data.

For peer review only

Background

Globally, diabetes mellitus (DM), characterized by raised blood glucose, is a leading cause of premature mortality and disability[1]. The global prevalence of DM has been on the rise over the past several decades[1 2]. Recent estimates reflect the global prevalence of diabetes has nearly doubled among adults aged 18 years and above, rising from 4.7% in 1980 to 8.5% in 2014[3]. This growing burden is most prominent in low- and middle-income countries[4] particularly the Indian sub-continent [5]. South and Southeast Asia accounts for close to one-fifth of all diabetes cases worldwide and the prevalence of diabetes in this region is projected to increase by 71% by 2035[6]. In Bangladesh, specifically, the International Diabetes Federation projects the prevalence of diabetes will increase to more than 50% in the next 15 years[6].

The increase in prevalence of diabetes among Bangladeshi adults over the past few decades has been documented: Based on a meta-analysis of studies conducted from 1995 – 2010, the prevalence of diabetes among Bangladeshi adults aged 30 years and above increased from 4% in 1995 to 2000 and 5% in 2001 to 2005 to 9% in 2006 to 2010[7]. Studies to assess the burden of diabetes has been conducted in Bangladesh in both urban and rural populations over the last decades[8]. However, national data on the prevalence of diabetes or raised blood glucose starting at age 18 years are currently unavailable[8-11]. These data are valuable for monitoring progress made towards one of the nine global non-communicable disease (NCD) targets of the year 2025, set forth by the World Health Organization's (WHO) NCD Global Monitoring Framework: To observe a 0% increase in age-standardized prevalence of raised blood glucose or diabetes among persons aged ≥18 years[12]. As such in response to the WHO Global Action Plan for the Prevention and Control of NCDs[13], descriptive epidemiological data on the burden of raised blood glucose among adults starting at 18 years are needed to monitor national progress of interventions implemented to reduce the burden of DM in Bangladesh[14-16]. Here, using a nationally representative sample, we present data on prevalence and determinants of raised blood glucose by various sociodemographic factors such

For peer review only - http://bmjopen.bmj.com/site/about/guidelines.xhtml

For peer review only

as age, sex, and area of residence among Bangladeshi adults aged 18 years and above, residing in both urban and rural areas of the country. Additionally, we explore treatment patterns, and control of raised blood glucose among participants in our sample of Bangladeshi adults.

Methods

Data for this analysis were collected as part of a national assessment of the burden of musculoskeletal disorders in Bangladesh conducted by investigators from the Bangabandhu Sheikh Mujib Medical University (BSMMU) with technical assistance from the WHO Country Office for Bangladesh, as previously described[17]. The study was a population-based cross-sectional study carried out from November to December 2015 and followed the WHO STEP-wise approach to Surveillance of NCD risk factors (STEPS)[18]. The target population of this survey was men and women aged ≥18 years residing in rural and urban areas of Bangladesh. The exclusion criteria included, tourists and the institutionalized, including residents of hospitals, prisons, nursing homes, and army barracks.

<u>Sampling Methods</u>

To obtain a population-based sample of Bangladesh, this survey adopted a multistage, geographically clustered, probability-based sampling approach. Population statistics were obtained using the updated national census conducted by Bangladesh Bureau of Statistics (BBS) in 2009 [19]. To obtain our primary sampling unit (PSU), we utilized the following geographical distribution described: In Bangladesh, there are seven divisions, which are the largest administrative units of the country. Each division is divided into several districts (*Zila*) and within each district, there are several sub-districts (*Upazila*). Within sub-districts, *mauzas* and *mahallas* (commonly known as neighborhoods or blocks) are the smallest units within defined territories in rural and urban areas respectively. *Mauzas* and *mahallas* were considered the PSU for the study's sampling approach. The households within the *mauzas* and *mahallas*

For peer review only - http://bmjopen.bmj.com/site/about/guidelines.xhtml

were the secondary sampling units. We utilized the BBS' definition of household which is as follows: "a dwelling in which persons either related or unrelated were living together and taking food from the same kitchen"[19].

The power analysis and sample size calculations were completed based on the standardized approach outlined in WHO STEPS methodology [18]. Using the WHO STEPS methodology, the minimum number of participants required was 296 in each group (rural males, rural females, urban males, and urban females). Assuming a design effect of 1.5 adjusted within cluster population homogeneity, the necessary sample size was 1776. We assumed a response rate of 90% and determined we would need to contact at least 1973 adults. For simplicity, our target sample size was 2000. Twenty PSUs (8 urban and 12 rural) were randomly selected from 7 divisions of the country, with the probability proportional to the population size of each division. In each PSU, 100 consecutive households were selected. The even numbered households were designated as a "male household" and odd numbered households as a "female household." Finally, one male or female was approached to participate from each respective household as designated.

Data collection

Through a structured survey, we collected data on the following topics: musculoskeletal disorders[20], health history, and demographic data such as age, area of residence, education, current (last 12 month) occupation, tobacco use and physical activity. Physical measurements such as height, weight, waist circumference, blood glucose levels, and blood pressure were collected. To measure blood glucose levels, we obtained random blood glucose samples [3], per the clinical guidelines of diabetes diagnostic criteria of Bangladesh[21]. Capillary blood samples were consistently taken from the index finger of the right arm using a glucometer, namely Accu-chek Advantage (Roche Diagnostics Division, Grenzacherstrasse, Switzerland).

Each participant's history of diabetes was assessed based on self-report. Specifically, participants were asked: 1. Have you ever been diagnosed with diabetes by a health care

For peer review only - http://bmjopen.bmj.com/site/about/guidelines.xhtml

professional? 2. If yes, are you receiving treatment for diabetes? Treatment history of diabetes was confirmed by prescription, including medicine strips or insulin injection vials, or medical record when possible. Medicine strips or injection vials were checked when possible. The questionnaire was translated from English to Bengali, adapted and validated as per standard procedure. Data collection procedures were standardized across study sites through coordinated training of field staff conducted by epidemiologists, study physicians, and WHO staff members.

Blood pressure was measured by a trained field interviewer using the LifeSource UA-767+ blood pressure monitor, as recommended by the WHO, and appropriately sized arm cuffs. Blood pressure measurements were consistently taken on each participant's right arm at the level of the heart and elbow-assisted. The initial measurement was performed after five minutes of rest. After two minutes, the second measurement was taken. The mean of these two blood pressure readings was utilized as the final blood pressure for each participant.

Following data collection, participants were scheduled a visit with the study research physician within the following five-days. The research physician assessed the participant's medical history, either through self-report or using medical records when possible. Study physicians examined each participant to confirm the results of data collection through classical symptom assessment. When necessary, the participant was also evaluated by the divisional investigator for a second opinion of relevant diagnoses.

<u>Outcome definitions</u>

Our primary outcome of interest was prevalence of raised blood glucose. We utilized the American Diabetes Association (ADA) guidelines to define a diagnosis of raised blood glucose using random or casual plasma glucose test and symptom review by the study physician [22]. An individual was considered to have raised blood glucose if the plasma glucose level was 11.1 mmol/L or higher with classic symptoms of hyperglycemia, and/or if they self-reported to take medication to control their diabetes. Our secondary outcome of interest was prevalence of self-

For peer review only - http://bmjopen.bmj.com/site/about/guidelines.xhtml

reported diabetes mellitus. An individual was categorized as diabetic if they self-reported to have been previously diagnosed with diabetes by a health care provider.

Covariates

The following variables were assessed as covariates for analysis: area of residence, sex, age, education, occupation, wealth index, body mass index (BMI), BP, and waist circumference. Education was categorized into four groups: no education, primary education (completed grade ≤5), secondary education (completed ≤ grade 10), and above secondary education (completed ≥ grade 12). Occupation was categorized into five groups. These groups included: professional employment (field staff, police officer, guard, doctor, engineer, professional, business man, desk job), unemployed or retired, industrial worker or day laborer, housewife, and other (shop keeper, weaver, driver, student, beggar, cook, carpenter, tailor, migrant workers and fishermen).

Data on physical activity was collected based on self-report. First, respondents were asked the number of days they engaged in in vigorous, moderate, or light physical activity throughout a typical week. The following definitions were used to define (1) vigorous, (2) moderate, and (3) light physical activity, respectively: (1)vigorous activity was defined as any activity that causes large increases in breathing or heart rate, if continued for at least 10 minutes (e.g. running, carrying heavy loads, digging or construction work); (2) Moderate activity was defined as any activity that causes small increase in breathing or heart rate, if continued for at least 10 minutes (brisk walking or carrying light loads); and (3) Light physical activity was defined as activities such as office work. Next, we asked participants to estimate how many minutes per day they engaged in the activity. MET-minute was calculated using the STEPS protocol[23] as follows: one minute of light activity was equivalent to 1 MET-minute; one minute in moderate-intensity activity related activities was equivalent to 4 MET-minutes, and one minute of vigorous-intensity was equivalent to 8 MET-minutes. Physical activity was then categorized based on total MET-minutes per week. Participants who spent 3000 or more MET-minutes per

For peer review only - http://bmjopen.bmj.com/site/about/guidelines.xhtml

week were categorized in the vigorous physical activity group, 600-3000 MET-minutes were categorized as moderate physical activity, and <600 MET-minutes were categorized as low physical activity.

The wealth index was constructed using principal component analysis. Asset information collected covered information on household ownership of nineteen items, including: electricity, flush toilet, land telephone, cell phone, television, radio, refrigerator, car, motorcycle, washing machine, bicycle, sewing machine, wardrobe, table, bed or cot, chair or bench, watch or clock. Additionally, we assessed the main type of material used to build each participant's home (i.e. cement, tin, bamboo, or thatched straw). Each asset was assigned a weight (factor score) generated through principal components analysis, and the resulting asset scores were standardized to a normal distribution with a mean of zero and standard deviation of one. Each household was then assigned a score for each asset, and the scores were summed up; individuals were ranked according to the total score of the household in which they resided. The sample was then divided into quartiles from quartile one (lowest) to quartile four (highest). Using height (centimeters) and weight (kilograms) measurements, we calculated BMI (height/weight^2). BMI was categorized in the following groups: underweight (≤ 18.5), normal (≤25), overweight (25.1-30) and obese (>30). Waist circumference was measured in centimeters (cm). Participants were categorized as abdominally obese if waist circumference was 90 cm and above for men, or 80 cm and above for women. Prehypertension was defined as SBP ≥120 mmHg but < 140 mmHg and/or DBP ≥ 80 mmHg but <90 mmHg and not taking anti-hypertensive medication at the time of the survey. We utilized the WHO's guidelines for cut-off points to define hypertension[24]. An individual was considered to have hypertension if systolic blood pressure (SBP) was ≥140 mmHg (millimeters of mercury) and/or, diastolic blood pressure (DBP) ≥90 mmHg, and/or taking anti-hypertensive medication based on self-report.

<u>Age-Standardized Prevalence Estimates</u>

For peer review only - http://bmjopen.bmj.com/site/about/guidelines.xhtml

To facilitate comparison of overall raised blood glucose among Bangladeshi adults across global populations with different age compositions we calculated age-standardized prevalence estimates with 95% confidence intervals (CIs) using the WHO's World Standard Population [25]. The World Standards database (WHO 2000-2025) provided population estimates for 18 and 19 age groups, as well as single year ages. To derive single ages from the 5-year age group proportions publically available, we used the Beers "Ordinary" Formula[26]. The following formula was utilized for standardization:

$\sum p_i * w_i / \sum w_i$, where p = observed prevalence and w = world population weight.

<u>Data Analysis</u>

Sociodemographic variables were presented with mean and standard deviation (SD) for continuous variables, and using proportions for categorical variables. For bivariate analyses, study participants were divided by sex and into four age groups (18-29, 30-44, 45-54 and ≥55 years). We calculated the prevalence of our primary outcome by key demographic variables and calculated 95% CIs using the binomial exact method.

To estimate determinants of raised blood glucose, we computed prevalence ratios with Poisson regression using robust estimation of standard errors[27-29]. Potential variables for inclusion in the model were assessed using prior published literature and bivariate Poisson regression analysis; an arbitrary p-value of <0.10 was used as criteria to include the variable in the multivariable Poisson regression model to control for confounding effects. For multivariable Poisson regression models, adjusted prevalence ratios (aPR), and 95% CIs for each independent variable were calculated. Additionally, *<0.05* was used as the level of significance. Multivariable Poisson regression models were generated separately for urban and rural participants to account for possible effect measure modification. Collinearity was assessed using the variance inflation factor to ensure a strong linear relationship among independent

For peer review only - http://bmjopen.bmj.com/site/about/guidelines.xhtml

variables included in the model was not present. All statistical procedures were performed using Stata/SE 15.0 (StataCorp LP, Texas, USA) software package.

Results

Background Characteristics

Of the 2000 adults approached, 1843 agreed to participate in our study leading to a response rate of 92.1%. Twenty-four female participants were pregnant and were dropped from subsequent analyses to ensure those with gestational diabetes were not included. There were 892 (49.0%) male and 927 (50.9%) female respondents (Table 1). Age of our participants ranged from 18 to 90 years. The mean age and education level of participants was 40.5 (SD=14.7) years and 5.7 (SD=5.1) years, respectively. The majority of the population was married (88.0%) and employed as either an industrial worker/day laborer (26.5%) or housewife (39.9%). Almost half of the population never used some form of tobacco. The majority of participants engaged in vigorous physical activity over an average week (69.3%). The mean BMI was 22.1 (SD = 4.1) and mean waist circumference was 78.4 cm (SD = 11.6). Overall, the mean systolic blood pressure was 116.1 mmHg (SD = 17.1) and diastolic blood pressure was 76.1 mmHg (SD=10.5). The mean blood glucose level was 6.4 mmol/L (SD = 2.4). Figure 1 presents the distribution of blood glucose levels by various demographic factors.

Prevalence and Risk Factors for Raised Blood Glucose

The prevalence of raised blood glucose was 5.5% (95% CI: 4.5-6.6). This prevalence was significantly higher among urban participants (9.8%, 95%CI: 7.7-12.2) than rural participants (2.8%, 95% CI: 1.9 – 3.9) (Table 2) and increased as age increased (Figure 2). The highest prevalence of raised blood glucose was observed among those aged ≥55 years, at 8.2% (95% CI: 4.6-13.1) among men and 9.4% (95% CI: 5.4-14.8) among women. The age-standardized prevalence of raised blood glucose was 5.6% (95% CI: 4.6 – 6.8). The age-

For peer review only - http://bmjopen.bmj.com/site/about/guidelines.xhtml

standardized prevalence of raised blood glucose among urban and rural residents was 10.5% (95% CI: 9.2-12.1) and 2.8% (95% CI: 2.1-3.7), respectively. Among men and women, the age-standardized prevalence of raised blood glucosewas 4.9% (95% CI: 3.9-6.0) and 6.0% (95% CI: 4.9-7.2), respectively.

Self-Reported Diabetes Mellitus and Raised Blood Glucose

Ninety-five participants (5.2%) self-reported to have been previously diagnosed with diabetes by a health care provider. However, 25 of these individuals did not meet our criteria of diagnosis ofraised blood glucose as they did not take medication to control their diabetes and their plasma glucose was below 11.1 mmol/L. Therefore, 69.3% of those with raised blood glucose, per the study definition, were previously diagnosed with diabetes by a health care provider. The proportion of men and women who were previously diagnosed with diabetes by a health care based on self-report was higher among urban residents (men: 8.4%; women: 9,1%) than among rural residents (men: 2.9%; women: 3.0%) (Figure 3a). However, overall, the large majority (81.8%) reported they did not know if they had been previously diagnosed with diabetes; this proportion was higher among rural residents (87.8%) than urban residents (72.2%). Among participants previously diagnosed with diabetes based on self-report, 72.6% reported to take medication to control their diabetes. Urban women more frequently (96.7%) self-reported to take diabetes medication than urban men (62.1%) (Figure 3b).

Among participants who were diagnosed with raised blood glucose at study measurement, over one-third (37.9%), of urban men self-reported to have diabetes, however, did not take any medication to control their diabetes. Among rural participants, the proportion of women who did not take medication to control their self-reported diabetes was higher (52.9%) than men (31.3%). Although three quarters of self-reported diabetic participants reported to take medication to control their diabetes, 31% continued to have high blood sugar levels indicating uncontrolled diabetes at study measurement (Figure 4).

For peer review only - http://bmjopen.bmj.com/site/about/guidelines.xhtml

Determinants of Raised Blood Glucose

Table 2 presents results of multivariable Poisson regression with robust variance analyses to identify determinants of raised blood glucose. Among urban participants, those of older age, lowest wealth quartile, hypertension, low physical activity, and with abdominal obesity based on waist circumference, were more likely to have raised blood glucose. The prevalence of raised blood glucose was significantly highest among those aged ≥55 years (aPR 3.92, 95% CI:1.48 – 10.39) compared to individuals aged 18-29 years of age. When compared to those in the 4th (highest) wealth quartile, urban residents in the first (lowest) wealth quartile had 3.18 times the prevalence of raised blood glucose. For urban individuals with hypertension, the prevalence of raised blood glucose was 2.65 (95% CI: 1.30-5.38) times that of individuals without hypertension. The prevalence of raised blood glucose among those with low physical activity was 3.01 (95% CI: 1.42- 6.38) times that of urban participants with vigorous physical activity. Abdominal obesity also significantly increased the prevalence of raised blood glucose among urban participants (aOR: 2.54, 95% CI: 1.35-4.77). Among rural participants, the only observed determinants of raised blood glucose were hypertension (aPR: 5.39, 95% CI: 1.94 – 14.96) and abdominal obesity (aPR: 2.95, 95% CI: 1.32 – 6.58).

Discussion

Using data from this nationally representative sample, we estimate that about one in twenty Bangladeshi adults aged ≥18 years have raised blood glucose. The prevalence of raised blood glucose was higher among urban residents (9.8%), than rural residents (2.8%). Prevalence of raised blood glucose increased with age, urban residence, abdominal obesity, low physical activity, and hypertension. As diabetes is characterized by raised blood glucose, targeting high-risk groups identified in this analysis could be prioritized for organized diabetes preventive programs in Bangladesh. Bangladesh has adopted the goals and targets set forth by

For peer review only - http://bmjopen.bmj.com/site/about/guidelines.xhtml

the WHO's Global Monitoring Framework for the Prevention and Control of NCDs for the year 2025[12]. One of these targets is to ensure there is a 0% increase in the age-standardized prevalence of raised blood glucose among adults aged ≥18 years by the year 2025. To our knowledge, the present study is the first to report national estimates on prevalence of raised blood glucose starting at age 18 years in both urban and rural areas of Bangladesh. Data gathered from this national-level study are critical towards the measurement of progress towards the nine global targets for Bangladesh for 2025.

In our study, the age-standardized prevalence of raised blood glucose was 5.6%, and was significantly higher in urban areas than rural areas. This may be due to differences in life style factors such as physical activity, diet, and tobacco use. These findings are similar to prior studies conducted to measure the prevalence of diabetes among adults in Bangladesh[8 10 11 30 31]. A recently published review, identified about 22 studies conducted to estimate the prevalence of diabetes in different settings, including rural and urban, and varying age groups including ≥20 years, ≥30 years, and ≥ 35 years[8]. Estimates of diabetes prevalence ranged from 4.5% and 35%, however, the pooled estimate was 7.4% (95% CI: 7.2-7.6). The pooled estimate is higher than our study's findings, however, our cohort is generally younger and this may lead to the lower prevalence estimate.

Significant determinants of raised blood gluocse in both urban and rural areas of our assessment included hypertension, low physical activity, and abdominal obesity. Interestingly, there was no association of diabetes identified for increasing body mass index (BMI). This indicates that abdominal obesity may be a more significant factor to consider than BMI. Prior studies conducted in Bangladesh have also identified a positive association of central (or abdominal) obesity with diabetes[32]. Interestingly, our assessment found a decrease in odds of diabetes with increasing wealth quartile. Prior studies have conflicting findings on the risk of diabetes and other NCDs, among the wealthy based on demographic features such as area of residence. One prior study conducted in Bangladesh found that people from the highest wealth

For peer review only - http://bmjopen.bmj.com/site/about/guidelines.xhtml

quintile are significantly more likely to have diabetes than people from the lowest wealth quintile[33]. However, another found a high burden of selected NCDs, including diabetes, among the lowest wealth quintile populations in rural areas and wealthy populations in urban areas[34]. Further study is warranted to assess the reliability of wealth indices as a measurement of socioeconomic status and wealth among Bangladeshi adults.

In our study, a high proportion (~70%) of those with raised blood glucose self-reported to have bene previously diagnosed with diabetes and therefore, aware of their condition. Additionally, 72% reported to take medication to control their diabetes. However, we found that almost one-third of those who self-reported to take medication for their diabetes, continued to have raised blood glucose.Efforts should be made to ensure diabetics in Bangladesh are treated for their condition and secondary prevention of complications of diabetes, such as diabetic retinopathy. This is of particular concern in developing countries where resources are limited and cost-effective solutions for chronic disease treatment should be prioritized. A recently published study found that healthcare expenditure in persons with diabetes in Bangladesh is six times higher than in persons without diabetes [15]. Prevention and management of diabetes is likely to be a cost-saving approach for Bangladesh through the utilization of community health workers adequately trained to effectively screen for, and identify, people with diabetes [35].

This study has several strengths. Data collected for our study was of a nationally representative sample indicating our results are highly generalizable to the total population of Bangladeshis aged 18 years and above. Our sample of 1843 allowed us to do an effective analysis of further subgroups.. However, several limitations should also be considered when interpreting the results of this analysis. We assessed blood glucose levels using random capillary blood samples, however, we did not assess history of classical symptoms of hyperglycemia, which is necessary to diagnose diabetes according to the ADA guidelines[22]. Additionally, we were unable to measure factors such as family history of diabetes, and obtain

For peer review only - http://bmjopen.bmj.com/site/about/guidelines.xhtml

blood glucose samples in fasting or two-hour post-prandial status to also assess prediabetes. Future studies should consider the addition of glycosylated hemoglobin measurement when assessing the prevalence of diabetes as this method could provide more long-term and stable diagnosis of diabetes mellitus. As such, we have an adequate sample size to measure prevalence of diabetes. Finally, due to the cross-sectional nature of this study, we were unable to define temporality of certain determinants of raised blood glucose identified and therefore, unable to assess causality.

Conclusion

Data from this nationally-representative sample of Bangladeshi adults aged 18 years and above will be critical to inform the progress of NCD control in Bangladesh per the WHO's Global Monitoring Framework and goals for 2025. We found that about one in twenty Bangladeshi adults aged ≥18 years have raised blood glucose. Among urban residents, we found that about one in ten Bangladeshi adults aged ≥18 years have raised blood glucose . Bangladeshi adults with hypertension and abdominal obesity are high-risk groups for the development of diabetes and should be targeted for routine screening for diabetes. Preventive methods such as lifestyle changes and medication should be recommended by primary care providers in Bangladesh to avoid the future development of CVDs among this group. In order to control the prevalence of raised blood glucose, and reduce the burden of diabetes and associated risk factors, national initiatives such as training community health workers to deliver primary care and implementing universal health coverage should be implemented to curb the spread of NCDs in Bangladesh.

For peer review only - http://bmjopen.bmj.com/site/about/guidelines.xhtml

Figures

Figure 1: Urban and Rural differences in distribution of blood glucose levels based on random capillary blood measurement among (A) All Participants, and (B) Men and Women (n = 1819)

Figure 2: Prevalence of raised blood glucose among Bangladeshi adults aged 18 years and above by sex and age group, 2015 (n = 1819)

Figure 3: Bangladeshi adults aged 18 years and above with (A) self-reported diabetes and (B) self-reported diabetics on diabetes medications, 2015

Figure 4: Self-reported diabetics aged 18 years or older who take diabetes medication with raised blood glucose on study measurement (≥11.0mmol/L)

For peer review only

Abbreviations

WHO: World Health Organization

ADA: American Diabetes Association

aPR: Adjusted prevalence ratio

BBS: Bangladesh Bureau of Statistics

BMI: Body mass index

BP: Blood pressure

BSMMU: Bangabandhu Sheikh Mujib Medical University

CI: Confidence interval

CVD: Cardiovascular disease

MET: Metabolic equivalent

mmHg: Millimeter of mercury

mmol/L: Millimoles per litre

NCD: Non-communicable disease

SBP: Systolic blood pressure

DBP: Diastolic blood pressure

PSU: Primary sampling unit

SD: Standard deviation

STEPS: STEPwise approach to surveillance

For peer review only

For peer review only - http://bmjopen.bmj.com/site/about/guidelines.xhtml

Footnotes

Ethics approval and consent to participate: Ethical guidelines as outlined by the Declaration of Helsinki were followed throughout the study. Ethical clearance was obtained from Institutional Review Board of Bangabandhu Sheikh Mujib Medical University (BSMMU). We obtained permission from the relevant administrative units of the surveyed districts. Orientations with community leaders (elected representatives of the local government offices) were conducted prior to data collection for community engagement in the study's implementation process. Written (or thumb impression if unable to write) consent was obtained from the respondents in Bangla as per BSMMU Institutional Review Board (IRB) guidelines.

Consent to publish: All authors consent to the publication of this manuscript.

Availability of data and materials: The datasets used and/or analyzed during the current study are available from the corresponding author on reasonable request.

Competing Interests: The authors declare no competing interests. The authors alone are responsible for views expressed in this article and they do not necessarily represent the views, decisions or policies of the institutions with which they are affiliated.

Funding: The study was conducted with technical and financial assistance of the World Health Organization Country Office for Bangladesh.

Authors contributions: JYI: analyzed data, interpreted results, and drafted the manuscript. MMZ: designed the study, interpreted results critically, conceptualized the manuscript, guided manuscript writing, and critically reviewed it. MRB, SAH, SA, ZAQ: trained the field team, implemented the survey, processed and analyzed data, and reviewed the manuscript.

Acknowledgements: The authors thank Mr. Hassanuzzaman Khan for his efforts on data management.

For peer review only - http://bmjopen.bmj.com/site/about/guidelines.xhtml

References

1. Lozano R, Naghavi M, Foreman K, et al. Global and regional mortality from 235 causes of death for 20 age groups in 1990 and 2010: a systematic analysis for the Global Burden of Disease Study 2010. *Lancet (London, England)* 2012;380(9859):2095-128. doi: 10.1016/S0140-6736(12)61728-0

2. Murray CJ, Vos T, Lozano R, et al. Disability-adjusted life years (DALYs) for 291 diseases and injuries in 21 regions, 1990-2010: a systematic analysis for the Global Burden of Disease Study 2010. *Lancet (London, England)* 2012;380(9859):2197-223. doi: 10.1016/S0140-6736(12)61689-4

3. Global report on diabetes. Geneva, Switzerland: World Health Organization 2016.

4. Wild S, Roglic G, Green A, et al. Global prevalence of diabetes: estimates for the year 2000 and projections for 2030. *Diabetes care* 2004;27(5):1047-53.

5. Jayawardena R, Ranasinghe P, Byrne NM, et al. Prevalence and trends of the diabetes epidemic in South Asia: a systematic review and meta-analysis. *BMC public health* 2012;12:380. doi: 10.1186/1471-2458-12-380

6. IDF diabetes atlas sixth edition Brussels, Belgium International Diabetes Federation, 2013.

7. Saquib N, Saquib J, Ahmed T, et al. Cardiovascular diseases and type 2 diabetes in Bangladesh: a systematic review and meta-analysis of studies between 1995 and 2010. *BMC public health* 2012;12:434. doi: 10.1186/1471-2458-12-434

8. Biswas T, Islam A, Rawal LB, et al. Increasing prevalence of diabetes in Bangladesh: a scoping review. *Public health* 2016;138:4-11. doi: 10.1016/j.puhe.2016.03.025

9. Rahman MS, Akter S, Abe SK, et al. Awareness, treatment, and control of diabetes in Bangladesh: a nationwide population-based study. *PloS one* 2015;10(2):e0118365. doi: 10.1371/journal.pone.0118365

For peer review only

For peer review only - http://bmjopen.bmj.com/site/about/guidelines.xhtml

10. Akter S, Rahman MM, Abe SK, et al. Prevalence of diabetes and prediabetes and their risk factors among Bangladeshi adults: a nationwide survey. *Bulletin of the World Health Organization* 2014;92(3):204-13, 13A. doi: 10.2471/BLT.13.128371

11. Zaman MM, Choudhury SR, Ahmed J, et al. Blood glucose and cholesterol levels in adult population of Bangladesh: Results from STEPS 2006 survey. *Indian heart journal* 2016;68(1):52-6. doi: 10.1016/j.ihj.2015.06.031

12. NCD Global Monitoring Framework: World Health Organization; 2017 [Available from: http://www.who.int/nmh/global_monitoring_framework/en/ accessed 09/29 2017.

13. Global action plan for the prevention and control of noncommunicable diseases 2013-2020. Geneva, Switzerland: World Health Organization, 2013.

14. Siddique MKB, Islam SMS, Banik PC, et al. Diabetes knowledge and utilization of healthcare services among patients with type 2 diabetes mellitus in Dhaka, Bangladesh. *BMC health services research* 2017;17(1):586. doi: 10.1186/s12913-017-2542-3

15. Shariful Islam SM, Lechner A, Ferrari U, et al. Healthcare use and expenditure for diabetes in Bangladesh. *BMJ Glob Health* 2017;2(1):e000033. doi: 10.1136/bmjgh-2016-000033

16. Haghparast-Bidgoli H, Shaha SK, Kuddus A, et al. Protocol of economic evaluation and equity impact analysis of mHealth and community groups for prevention and control of diabetes in rural Bangladesh in a three-arm cluster randomised controlled trial. *BMJ open* 2018;8(8):e022035. doi: 10.1136/bmjopen-2018-022035

17. Islam JY, Zaman MM, Haq SA, et al. Epidemiology of hypertension among Bangladeshi adults using the 2017 ACC/AHA Hypertension Clinical Practice Guidelines and Joint National Committee 7 Guidelines. *Journal of human hypertension* 2018;32(10):668-80. doi: 10.1038/s41371-018-0087-5

18. Chronic Disease and Health Promotion: STEP wise approach to surveillance (STEPS). Geneva: World Health Organization.

For peer review only

For peer review only - http://bmjopen.bmj.com/site/about/guidelines.xhtml

19. Population and Housing Census 2001, updated in 2009. Dhaka, Bangladesh: Bangladesh Bureau of Statistics 2009.

20. Goycochea-Robles MV, Sanin LH, Moreno-Montoya J, et al. Validity of the COPCORD core questionnaire as a classification tool for rheumatic diseases. *J Rheumatol Suppl* 2011;86:31-5. doi: 10.3899/jrheum.100955

21. Guidelines for Care of Type 2 Diabetes Mellitus in Bangaldesh. Dhaka, Bangladesh: BIRDEM Clinical Research Group, 2003.

22. American Diabetes A. 2. Classification and Diagnosis of Diabetes. *Diabetes care* 2017;40(Suppl 1):S11-S24. doi: 10.2337/dc17-S005

23. Moniruzzaman M, Mostafa Zaman M, Islalm MS, et al. Physical activity levels in Bangladeshi adults: results from STEPS survey 2010. *Public health* 2016;137:131-8. doi: 10.1016/j.puhe.2016.02.028

24. A global brief on Hypertension: Silent Killer, global public health crisis. Geneva, Switzerland: World Health Organization, 2013.

25. Ahmad OB, Boschi-Pinto C, Lopez AD, et al. Age Standardization of Rates: A New WHO Standard. GPE Discussion Paper Series: No31 Geneva, Switzerland: World Health Organization, 2001.

26. World (WHO 2000-2025) Standard Rockville, MD: National Cancer Institute: Surveillance, Epidemiology, and End Results Program; 2017 [Available from: https://seer.cancer.gov/stdpopulations/world.who.html accessed December 17 2017.

27. Coutinho LM, Scazufca M, Menezes PR. Methods for estimating prevalence ratios in cross-sectional studies. *Revista de saude publica* 2008;42(6):992-8.

28. Behrens T, Taeger D, Wellmann J, et al. Different methods to calculate effect estimates in cross-sectional studies. A comparison between prevalence odds ratio and prevalence ratio. *Methods Inf Med* 2004;43(5):505-9.

For peer review only - http://bmjopen.bmj.com/site/about/guidelines.xhtml

For peer review only

29. Barros AJ, Hirakata VN. Alternatives for logistic regression in cross-sectional studies: an empirical comparison of models that directly estimate the prevalence ratio. *BMC medical research methodology* 2003;3:21. doi: 10.1186/1471-2288-3-21

30. Hussain A, Rahim MA, Azad Khan AK, et al. Type 2 diabetes in rural and urban population: diverse prevalence and associated risk factors in Bangladesh. *Diabetic medicine : a journal of the British Diabetic Association* 2005;22(7):931-6. doi: 10.1111/j.1464-5491.2005.01558.x

31. Rahman M, Zaman MM, Islam JY, et al. Prevalence, treatment patterns, and risk factors of hypertension and pre-hypertension among Bangladeshi adults. *Journal of human hypertension* 2017 doi: 10.1038/s41371-017-0018-x

32. Alam DS, Talukder SH, Chowdhury MA, et al. Overweight and abdominal obesity as determinants of undiagnosed diabetes and pre-diabetes in Bangladesh. *BMC Obes* 2016;3:19. doi: 10.1186/s40608-016-0099-z

33. Tareque MI, Koshio A, Tiedt AD, et al. Are the rates of hypertension and diabetes higher in people from lower socioeconomic status in Bangladesh? Results from a nationally representative survey. *PloS one* 2015;10(5):e0127954. doi: 10.1371/journal.pone.0127954

34. Biswas T, Islam MS, Linton N, et al. Socio-Economic Inequality of Chronic Non-Communicable Diseases in Bangladesh. *PloS one* 2016;11(11):e0167140. doi: 10.1371/journal.pone.0167140

35. Gaziano TA, Abrahams-Gessel S, Denman CA, et al. An assessment of community health workers' ability to screen for cardiovascular disease risk with a simple, non-invasive risk assessment instrument in Bangladesh, Guatemala, Mexico, and South Africa: an observational study. *The Lancet Global health* 2015;3(9):e556-63. doi: 10.1016/S2214-109X(15)00143-6

For peer review only - http://bmjopen.bmj.com/site/about/guidelines.xhtml

Table 1: Background Characteristics of Bangladeshi adult participants, 2015 (n = 1819)

	Total (n = 1819)			Urban (n = 708)			Rural (n = 1111)		
Characteristic	Mean (SD)	n	%	Mean (SD)	n	%	Mean (SD)	n	%
Gender									
Male		892	49.0		345	48.7		547	49.2
Female		927	50.9		363	51.3		564	50.8
Age(years)	40.5 (14.7)			39.1 (13.9)			41.4 (15.1)		
Education (Years) [a]	5 (0 - 9)			8 (3-12)			4 (0-8)		
Marital Status									
Never Married		110	6.1		54	7.6		56	4.9
Married		1601	88.0		619	87.4		982	88.4
Separated/Divorced/Widowed		108	5.9		35	4.9		73	6.5
Occupation									
Professional employment [b]		279	15.2		189	26.7		90	8.1
Unemployed/retired		98	5.3		43	6.1		55	4.9
Industrial worker/Day Laborer		483	26.6		120	16.9		363	32.7
Housewife		726	39.9		247	34.9		479	43.2
Other [c]		232	12.8		109	15.4		123	11.1
Wealth Index [d]									
1st Wealth Quartile		407	22.4		110	15.5		297	26.7
2nd Wealth Quartile		533	29.3		171	24.2		362	32.6
3rd Wealth Quartile		429	23.6		179	25.3		250	22.5
4th Wealth Quartile		450	24.7		248	35.0		202	18.2
Tobacco Use [e]									
Never		859	47.2		389	54.9		470	42.3
Current Use		821	45.1		268	37.9		553	49.8
Past Use		139	7.6		51	7.2		88	7.9
Smoking Tobacco Use [f]									
Every day / Occasionally		494	27.2		169	23.9		325	29.3
Past Use		104	5.7		38	5.4		66	5.9
Never		1221	67.1		501	70.8		720	64.8
Smokeless Tobacco Use [g]									
Every day / Occasionally		529	29.1		150	21.2		379	34.1
Past Use		51	2.8		21	2.9		30	2.7
Never		1239	68.1		537	75.9		702	63.2
Physical Activity [h]									
Vigorous		1268	69.7		445	62.9		823	74.1
Moderate		464	25.5		234	33.1		230	20.7
Low		87	4.7		29	4.1		58	5.2

For peer review only

For peer review only - http://bmjopen.bmj.com/site/about/guidelines.xhtml

Body Mass Index [i]	22.1 (4.1)	23.3 (4.5)	21.3 (3.7)
Waist Circumference (cm)	78.4 (11.6)	81.8 (12.6)	76.2 (10.3)
Blood Pressure			
Systolic Blood Pressure (mmHg)	116.1 (17.1)	117.9 (16.6)	115.0 (17.3)
Diastolic Blood Pressure (mmHg)	76.1 (10.5)	77.9 (10.9)	74.9 (10.1)
Blood Glucose Level (mmol/l)	6.4 (2.4)	6.6 (2.9)	6.3 (2.1)

Abbreviations: SD, standard deviation

[a] Calculated median and interquartile range for education as the data are skewed

[b] Professional occupation includes: Field staff, police officer, guard, doctor, engineer, professional, business man, desk job

[c] Other occupation includes: Shop keeper, weavers, driver, student, beggar, cook, carpenter, tailor, migrant workers and fishermen

[d] Wealth index was calculated using principal component analysis using data collected on household ownership of the following items: electricity, flushable toilet, land phone, cell phone, television, radio, refrigerator, private car, motor cycle, washing machine, bicycle, sewing machine, almirah/wardrobe, table, bed, chair/bench, watch/clock, as well as, type of main material used to build their homes roof, walls and floor

[e] Includes both smokeless tobacco and smoke tobacco

[f] Smoking tobacco use includes cigarettes, biri, hookah, etc.

[g] Smokeless tobacco use includes jodda, paan, white leaf, etc.

[h] Measured in MET-minutes; 1 MET stands for the amount of oxygen you consume and the number of calories you burn at rest.

[i] Body mass index (BMI) calculated by weight in kilogram divided by height in meter squared

For peer review only

Table 2: Determinants of raised blood glucose among Bangladeshi adults, 2015 (n = 1819)

	Total (n = 1819)		Urban (n = 708)		Rural (n = 1111)	
	Diabetes [a] Prevalence %	Adjusted PR [b] (95% CI)	Diabetes [a] Prevalence %	Adjusted OR [b] (95% CI)	Diabetes [a] Prevalence %	Adjusted OR [b] (95% CI)
**Characteristic**						
Area						
Urban	9.8	Ref.	-	-	-	-
Rural	2.8	0.44 (0.28 – 0.68)	-	-	-	-
Gender						
Male	4.9	Ref.	7.5	Ref.	3.3	Ref.
Female	5.9	1.05 (0.71 – 1.54)	11.9	1.26 (0.80 – 1.99)	2.2	0.61 (0.29 – 1.28)
Age (years)						
18 - 29	2.5	Ref.	2.5	Ref.	2.5	Ref.
30 - 44	4.9	1.48 (0.78 – 2.79)	8.4	2.55 (1.01 – 6.41)	2.5	0.76 (0.30 – 1.93)
45 - 54	7.4	2.18 (1.10– 4.31)	15.5	4.38 (1.62 – 11.59)	2.8	0.67 (0.25 – 1.83)
≥ 55	8.8	1.92 (0.95 – 3.86)	19.3	3.92 (1.48 – 10.39)	3.4	0.53 (0.17 – 1.67)
Educational Status						
No Education	3.4	Ref.	7.0	Ref.	2.1	Ref.
Primary Education	3.7	1.00 (0.53 – 1.86)	7.6	1.00 (0.44 – 2.30)	2.1	0.84 (0.30 – 2.30)
Secondary Education	6.9	1.67 (0.95 – 2.93)	12.1	1.94 (0.95 – 3.97)	2.9	1.01 (0.40 – 2.54)
Above Secondary Education	9.8	1.48 (0.77 – 2.84)	10.4	1.54 (0.71 – 3.33)	8.1	2.24 (0.68 – 7.41)
Wealth Index [c]						
1st Wealth Quartile	7.7	2.58 (1.57 – 4.24)	18.9	3.18 (1.80 – 5.62)	3.6	1.37 (0.53 – 3.49)
2nd Wealth Quartile	4.3	1.23 (0.71 – 2.14)	9.4	1.50 (0.82 – 2.77)	1.9	0.70 (0.24 – 2.05)
3rd Wealth Quartile	3.7	0.86 (0.47 – 1.58)	6.0	0.96 (0.48 – 1.92)	2.0	0.55 (0.18 – 1.73)
4th Wealth Quartile	6.6	Ref.	8.8	Ref.	3.9	Ref.
Blood Pressure						
Normal Blood Pressure	1.9	Ref.	3.5	Ref.	1.1	Ref.
Pre-Hypertension [d]	5.7	1.74 (1.00 – 3.01)	8.3	1.37 (0.69 – 2.74)	3.8	2.32(0.91 – 5.92)
Hypertension [e]	18.9	3.57 (2.01 – 6.34)	27.4	2.65 (1.30 – 5.38)	8.8	5.39(1.94 –14.96)
Physical Activity						
Vigorous	3.9	Ref.	6.9	Ref.	2.3	Ref.
Moderate	8.2	1.18 (0.78 – 1.77)	13.8	1.22 (0.77 – 1.93)	2.1	0.65 (0.22 – 1.88)
Low	14.9	3.04 (1.69 – 5.47)	20.7	3.01 (1.42 – 6.38)	12.1	2.58 (0.95 – 7.04)
Body Mass Index [f]						
Underweight (<18.5)	1.1	0.37 (0.12 – 1.13)	1.8	0.38 (0.07 – 1.95)	0.7	0.42 (0.09 – 2.02)

For peer review only - http://bmjopen.bmj.com/site/about/guidelines.xhtml

Normal (18.5 - 25)	4.7	Ref.	8.2	Ref.	2.8	Ref.
Overweight (25.1 - 30)	10.4	1.06 (0.68 – 1.65)	14.0	1.05 (0.61 – 1.80)	6.2	1.22 (0.51 – 2.91)
Obese (>30)	20.9	1.49 (0.87 – 2.57)	26.5	1.46 (0.81 – 2.64)	5.6	0.95 (0.12 – 7.60)
Waist Circumference (cm)						
Normal [g]	2.3	Ref.	3.8	Ref.	1.6	Ref.
Abdominally Obese [h]	13.3	2.49 (1.53 – 4.07)	18.1	2.54 (1.35 – 4.77)	6.8	2.95 (1.32 – 6.58)

Abbreviations: PR = prevalence ratio, CI = confidence intervals, Ref = referent category

[a] Raised blood glucose was defined as a capillary blood glucose level greater than or equal to 11.1 mmol/L or self-reported diabetes medication use

[b] Model adjusted for all variables included in table: sex, age, education, wealth index, blood pressure, body mass index, self-reported physical activity, and waist circumference

[c] Wealth index was calculated using principal component analysis using data collected on household ownership of the following items: electricity, flushable toilet, land phone, cell phone, television, radio, refrigerator, private car, motor cycle, washing machine, bicycle, sewing machine, almirah/wardrobe, table, bed, chair/bench, watch/clock, as well as, type of main material used to build their homes roof, walls and floor

[d] Pre-Hypertension was defined as SBP ≥120 mmHg but < 140 mmHg and/or DBP ≥ 80 mmHg but <90 mmHg and not taking anti-hypertensive medication at the time of the survey

[e] Hypertension was defined as systolic blood pressure (SBP) was ≥140 mmHg (millimeters of mercury) and/or, diastolic blood pressure (DBP) ≥90 mmHg and/or taking any-hypertensive medication

[f] Body mass index (BMI) calculated by weight in kilogram divided by height in meter squared

[g] Defined as <90 cm M; <80 cm F

[h] Defined as ≥90 cm M; ≥80 cm F

For peer review only

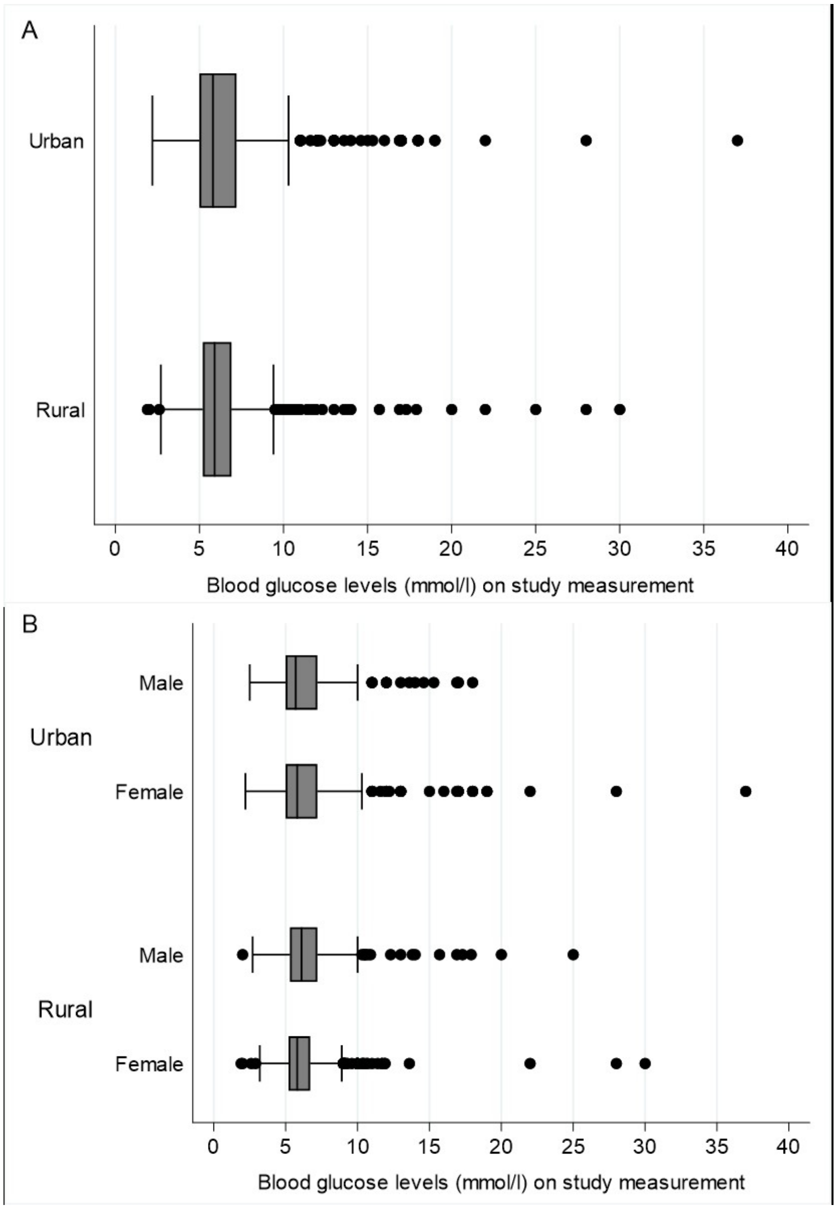

Figure 1: Urban and Rural differences in distribution of blood glucose levels based on random capillary blood measurement among (A) All Participants, and (B) Men and Women (n = 1819)

140x202mm (150 x 150 DPI)

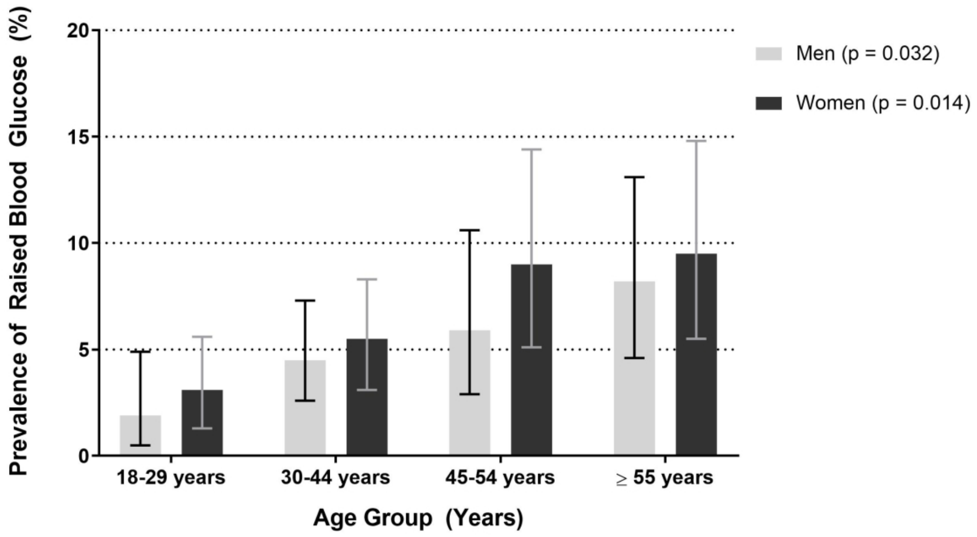

Figure 2: Prevalence of raised blood glucose among Bangladeshi adults aged 18 years and above by sex and age group, 2015 (n = 1819)

209x117mm (300 x 300 DPI)

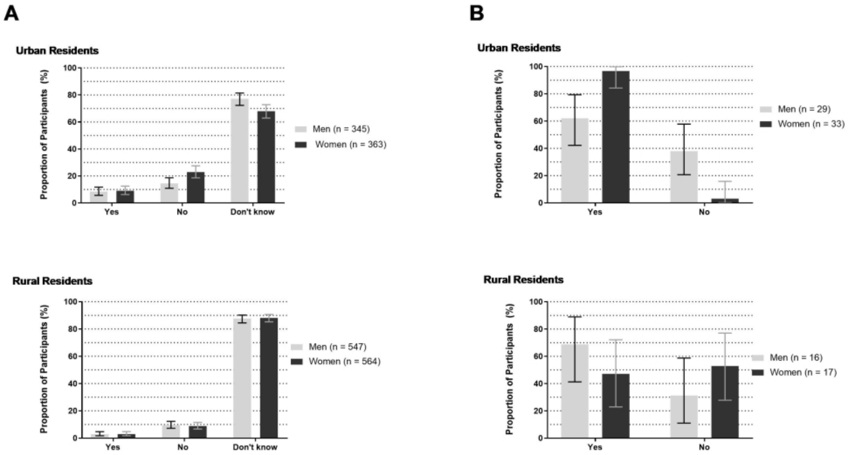

Figure 3: Bangladeshi adults aged 18 years and above with (A) self-reported diabetes and (B) self-reported diabetics on diabetes medications, 2015

245x120mm (300 x 300 DPI)

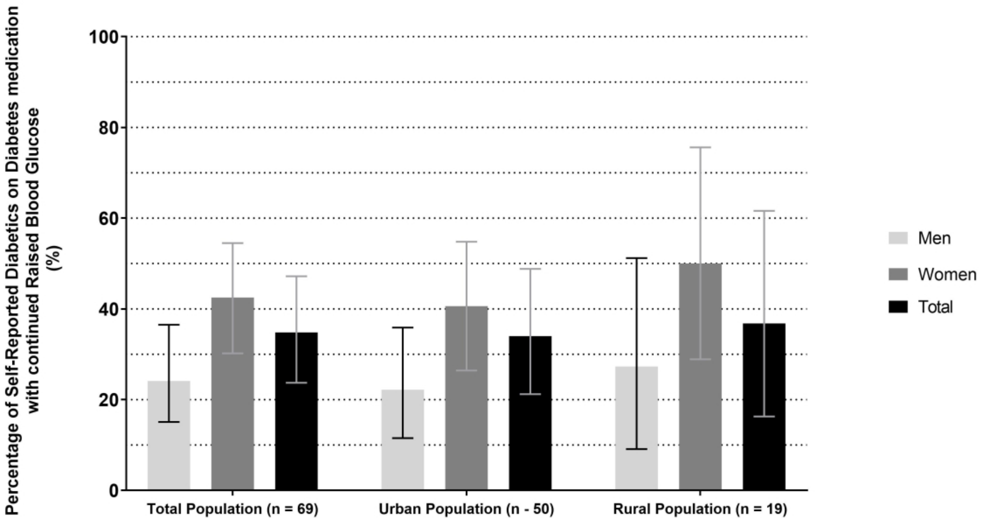

Figure 4: Self-reported diabetics aged 18 years or older who take diabetes medication with raised blood glucose on study measurement (≥11.0mmol/L)

286x154mm (300 x 300 DPI)

STROBE 2007 (v4) Statement—Checklist of items that should be included in reports of *cross-sectional studies*

Section/Topic	Item #	Recommendation	Reported on page #
**Title and abstract**	1	(*a*) Indicate the study's design with a commonly used term in the title or the abstract	2
		(*b*) Provide in the abstract an informative and balanced summary of what was done and what was found	2-3
**Introduction**			
Background/rationale	2	Explain the scientific background and rationale for the investigation being reported	5-6
Objectives	3	State specific objectives, including any prespecified hypotheses	6
**Methods**			
Study design	4	Present key elements of study design early in the paper	6
Setting	5	Describe the setting, locations, and relevant dates, including periods of recruitment, exposure, follow-up, and data collection	7
Participants	6	(*a*) Give the eligibility criteria, and the sources and methods of selection of participants	6
Variables	7	Clearly define all outcomes, exposures, predictors, potential confounders, and effect modifiers. Give diagnostic criteria, if applicable	8-9
Data sources/ measurement	8*	For each variable of interest, give sources of data and details of methods of assessment (measurement). Describe comparability of assessment methods if there is more than one group	7-10
Bias	9	Describe any efforts to address potential sources of bias	6-7 & 11
Study size	10	Explain how the study size was arrived at	7
Quantitative variables	11	Explain how quantitative variables were handled in the analyses. If applicable, describe which groupings were chosen and why	9-10
Statistical methods	12	(*a*) Describe all statistical methods, including those used to control for confounding	11
		(*b*) Describe any methods used to examine subgroups and interactions	11
		(*c*) Explain how missing data were addressed	N/A
		(*d*) If applicable, describe analytical methods taking account of sampling strategy	N/A
		(*e*) Describe any sensitivity analyses	N/A
**Results**			

For peer review only

Participants	13*	(a) Report numbers of individuals at each stage of study—eg numbers potentially eligible, examined for eligibility, confirmed eligible, included in the study, completing follow-up, and analysed	7
		(b) Give reasons for non-participation at each stage	7
		(c) Consider use of a flow diagram	N/A
Descriptive data	14*	(a) Give characteristics of study participants (eg demographic, clinical, social) and information on exposures and potential confounders	12
		(b) Indicate number of participants with missing data for each variable of interest	N/A
Outcome data	15*	Report numbers of outcome events or summary measures	
Main results	16	(*a*) Give unadjusted estimates and, if applicable, confounder-adjusted estimates and their precision (eg, 95% confidence interval). Make clear which confounders were adjusted for and why they were included	12
		(*b*) Report category boundaries when continuous variables were categorized	9
		(*c*) If relevant, consider translating estimates of relative risk into absolute risk for a meaningful time period	N/A
Other analyses	17	Report other analyses done—eg analyses of subgroups and interactions, and sensitivity analyses	11
**Discussion**			
Key results	18	Summarise key results with reference to study objectives	12-14
Limitations	19	Discuss limitations of the study, taking into account sources of potential bias or imprecision. Discuss both direction and magnitude of any potential bias	17-18
Interpretation	20	Give a cautious overall interpretation of results considering objectives, limitations, multiplicity of analyses, results from similar studies, and other relevant evidence	15-18
Generalisability	21	Discuss the generalisability (external validity) of the study results	17
**Other information**			
Funding	22	Give the source of funding and the role of the funders for the present study and, if applicable, for the original study on which the present article is based	20

*Give information separately for cases and controls in case-control studies and, if applicable, for exposed and unexposed groups in cohort and cross-sectional studies.

Note: An Explanation and Elaboration article discusses each checklist item and gives methodological background and published examples of transparent reporting. The STROBE checklist is best used in conjunction with this article (freely available on the Web sites of PLoS Medicine at http://www.plosmedicine.org/, Annals of Internal Medicine at http://www.annals.org/, and Epidemiology at http://www.epidem.com/). Information on the STROBE Initiative is available at www.strobe-statement.org.

For peer review only

BMJ Open

Prevalence and determinants of hyperglycemia among adults in Bangladesh: results from a population-based national survey

Journal:	*BMJ Open*
Manuscript ID	bmjopen-2019-029674.R1
Article Type:	Research
Date Submitted by the Author:	17-May-2019
Complete List of Authors:	Islam, Jessica; World Health Organization Country Office for Bangladesh, ; University of North Carolina at Chapel Hill Gillings School of Global Public Health, Department of Epidemiology<br>Zaman, MM; World Health Organization, Dhaka, Bangladesh,<br>Bhuiyan, Mahfuz; World Health Organization Country Office for Bangladesh<br>Haq, Syed; Bangabandhu Sheikh Mujib Medical University, Rheumatology<br>Ahmed, Shamim; Bangabandhu Sheikh Mujib Medical University<br>Al-Qadir, Zahid; Bangabandhu Sheikh Mujib Medical University
**Primary Subject Heading**:	Epidemiology
Secondary Subject Heading:	Diabetes and endocrinology, Global health
Keywords:	non-communicable diseases, chronic disease, raised blood glucose, Bangladesh

SCHOLARONE™
Manuscripts

Prevalence and determinants of hyperglycemia among adults in Bangladesh: results from a population-based national survey

Jessica Y. Islam[1,2], M. Mostafa Zaman*[2], Mahfuzur Rahman Bhuiyan[2], Syed Atiqul Haq[3], Shamim Ahmed[3], Zahid- Al- Quadir[3]

[1]Department of Epidemiology, UNC Gillings School of Global Public Health, Chapel Hill, NC; Email: islamjy@email.unc.edu

[2]World Health Organization, Dhaka, Bangladesh; Email: zamanm@who.int; Email: mahfuzdoc@gmail.com

[3]Department of Rheumatology, Bangabandhu Sheikh Mujib Medical University, Shahbag, Dhaka, Bangladesh. Email: haqsyedatiqul@gmail.com; Email: dr_shamim_ahmed@yahoo.com; Email: drzahid1981@yahoo.com

Corresponding Author:
Jessica Yasmine Islam, MPH
The University of North Carolina at Chapel Hill
Gillings School of Global Public Health
Department of Epidemiology
Chapel Hill, NC, USA 27599-7400
Email: islamjy@email.unc.edu

Manuscript word count: 4,200

For peer review only

For peer review only - http://bmjopen.bmj.com/site/about/guidelines.xhtml

Abstract

Objectives: With the increasing burden of non-communicable diseases in low- and middle-income countries, biological risk factors such as hyperglycemia are a major public health concern in Bangladesh. Hyperglycemia is an excess of glucose in the bloodstream and is often associated with type-2 diabetes mellitus. Nationally representative data of hyperglycemia prevalence starting from age ≥18 years are currently unavailable for Bangladeshi adults. The objective of this study was to assess the prevalence and determinants of hyperglycemia among adults in Bangladesh aged ≥18 years.

Study Design: Cross-sectional, population-based study

Setting and Participants: Data for this analysis were collected from a population-based nationally representative sample of 1843 adults, aged ≥18 years, from both urban and rural areas of Bangladesh. Demographic information, capillary blood glucose, blood pressure, height, weight, waist circumference, and treatment history were recorded.

Primary Outcome Measures: Hyperglycemia was defined as a random capillary blood glucose level of ≥11.1mmol/L (i.e. in the diabetic range) or currently taking medication to control type-2 diabetes, based on self-report.

Results: Overall, the prevalence of hyperglycemia was 5.5% (95% CI: 4.5-6.6) and was significantly higher among urban (9.8%, 95% CI: 7.7-12.2) than rural residents (2.8%, 95% CI: 1.9-3.9). The age-standardized prevalence of hyperglycemia was 5.6% (95% CI: 4.6-6.8). Among both urban and rural residents, the associated determinants of hyperglycemia included hypertension and abdominal obesity. About 5% of the total population self-reported to have

For peer review only - http://bmjopen.bmj.com/site/about/guidelines.xhtml

For peer review only

been previously diagnosed with type-2 diabetes; among these adults, over 25% were not taking medications to control their diabetes.

Conclusions: Our study found that about 1 out of 20 Bangladeshi adults aged ≥18 years have hyperglycemia. To control and prevent the development of type-2 diabetes, data from this study can be used to inform public health programming and provide descriptive information on surveillance of progress towards controlling diabetes in Bangladesh.

Keywords: non-communicable diseases, Bangladesh, hyperglycemia, diabetes, chronic disease

For peer review only

Article Summary

Strengths and Limitations of the Study

- This study utilized a multistage, geographically clustered, probability-based sampling approach to produce nationally representative data for Bangladesh.

- Currently, nationally representative data for the prevalence of hyperglycemia in the diabetic range is unavailable for adults aged 18-29 years. A strength of our study is that we included Bangladeshi adults aged 18 years and above to obtain novel data on the prevalence of hyperglycemia, and relevant non-communicable disease risk factors

- We were able to estimate the prevalence of hyperglycemia using capillary blood glucose measured at random. However, we were unable to measure the prevalence of prediabetes and diabetes as we did not obtain blood sugar levels using standardized methods, such as fasting blood glucose or 2-hour post-prandial measurements.

- Due to the cross-sectional nature of the study design, we were unable to assess temporality of risk factors identified and our outcomes of interest.

- We assessed type-2 diabetes medication history based on self-report and we were able to obtain medicine strips or vials records or prescription records of participants to confirm the self-reported data.

For peer review only - http://bmjopen.bmj.com/site/about/guidelines.xhtml

Background

Globally, diabetes mellitus (DM), characterized by hyperglycemia, is a leading cause of premature mortality and disability. Globally, almost half of all deaths attributable to high blood glucose occur before the age of 70 years, and the WHO estimates that diabetes was the seventh leading cause of mortality in 2016. The global prevalence of DM has been on the rise over the past several decades[1 2]. Estimates reflect the global prevalence of diabetes has nearly doubled among adults aged 18 years and above, rising from 4.7% in 1980 to 8.5% in 2014[3]. This growing burden is most prominent in low- and middle-income countries particularly the Indian sub-continent [4], which accounts for close to one-fifth of all diabetes cases worldwide. The prevalence of diabetes in this region is projected to increase by 71% by 2035[5]. In Bangladesh, specifically, the International Diabetes Federation projects the prevalence of diabetes will increase to more than 50% in the next 15 years[5].

The increasing prevalence of diabetes among Bangladeshi adults over the past few decades has been documented: Based on a meta-analysis of studies conducted from 1995 – 2010, the prevalence of diabetes among Bangladeshi adults aged 30 years and above increased from 4% in 1995 to 2000 and 5% in 2001 to 2005 to 9% in 2006 to 2010[6]. Studies to assess the burden of diabetes have been conducted in Bangladesh in both urban and rural populations over the last decades[7]. However, national data on the prevalence of diabetes or hyperglycemia in the diabetic range starting at age 18 years are currently unavailable[7-10]. These data are valuable for monitoring progress made towards one of the nine global non-communicable disease (NCD) targets of the year 2025, set forth by the World Health Organization's (WHO) NCD Global Monitoring Framework: To observe a 0% increase in age-standardized prevalence of hyperglycemia or diabetes among persons aged ≥18 years[11]. As such in response to the WHO Global Action Plan for the Prevention and Control of NCDs[12], descriptive epidemiological data on the burden of hyperglycemia among adults starting at 18 years are needed to monitor the national progress of interventions implemented to reduce the

For peer review only - http://bmjopen.bmj.com/site/about/guidelines.xhtml

burden of DM in Bangladesh[13-15]. Here, using a nationally representative sample, we present data on prevalence and determinants of hyperglycemia by various sociodemographic factors such as age, sex, and area of residence among Bangladeshi adults aged 18 years and above, residing in both urban and rural areas of the country. Additionally, we explore treatment patterns and control of hyperglycemia among participants in our sample of Bangladeshi adults.

Methods

Data for this analysis were collected as part of a national assessment of the burden of musculoskeletal disorders in Bangladesh conducted by investigators from the Bangabandhu Sheikh Mujib Medical University (BSMMU) with technical assistance from the WHO Country Office for Bangladesh, as previously described[16]. The study was a population-based cross-sectional study carried out from November to December 2015 and followed the WHO STEP-wise approach to Surveillance of NCD risk factors (STEPS)[17]. The target population of this survey was men and women aged ≥18 years residing in rural and urban areas of Bangladesh. The exclusion criteria included tourists and the institutionalized, including residents of hospitals, prisons, nursing homes, and army barracks.

<u>Sampling Methods</u>

To obtain a population-based sample of Bangladesh, this survey adopted a multistage, geographically clustered, probability-based sampling approach. Population statistics were obtained using the updated national census conducted by the Bangladesh Bureau of Statistics (BBS) in 2009 [18]. To obtain our primary sampling unit (PSU), we utilized the following geographical distribution described: In Bangladesh, there are seven divisions, which are the largest administrative units of the country. Each division is divided into several districts (*Zila*) and within each district, there are several sub-districts (*Upazila*). Within sub-districts, *mauzas* and *mahallas* (commonly known as neighborhoods or blocks) are the smallest units within

For peer review only - http://bmjopen.bmj.com/site/about/guidelines.xhtml

defined territories in rural and urban areas respectively. *Mauzas* and *mahallas* were considered the PSU for the study's sampling approach. The households within the *mauzas* and *mahallas* were the secondary sampling units. We utilized the BBS' definition of a household which is as follows: "a dwelling in which persons either related or unrelated were living together and taking food from the same kitchen"[18].

Sample size estimation

The power analysis and sample size calculations were completed based on the standardized approach outlined in the WHO STEPS methodology [17]. Using the WHO STEPS methodology, the minimum number of participants required was 296 in each group (rural males, rural females, urban males, and urban females). Assuming a design effect of 1.5 adjusted within cluster population homogeneity, the necessary sample size was 1776. We assumed a response rate of 90% and determined we would need to contact at least 1973 adults. For simplicity, our target sample size was 2000. Twenty PSUs (8 urban and 12 rural) were randomly selected from 7 divisions of the country, with the probability proportional to the population size of each division. In each PSU, 100 consecutive households were selected. The even numbered households were designated as a "male household" and odd numbered households as a "female household." Finally, one male or female was approached to participate from each respective household as designated.

Data collection

Through a structured survey, we collected data on the following topics: musculoskeletal disorders[19], health history, and demographic data such as age, area of residence, education, current (last 12 months) occupation, tobacco use, and physical activity. Physical measurements such as height, weight, waist circumference, blood glucose levels, and blood pressure were collected. To measure blood glucose levels, we obtained random blood glucose samples [3], per

For peer review only - http://bmjopen.bmj.com/site/about/guidelines.xhtml

For peer review only

the clinical guidelines of diabetes diagnostic criteria of Bangladesh[20]. Capillary blood samples were consistently taken from the index finger of the right arm using a glucometer, namely Accu-chek Advantage (Roche Diagnostics Division, Grenzacherstrasse, Switzerland).

Each participant's history of diabetes was assessed based on self-report. Specifically, participants were asked: 1. Have you ever been diagnosed with diabetes by a health care professional? 2. If yes, are you receiving treatment for diabetes? Treatment history of diabetes was confirmed by prescription, including medicine strips or insulin injection vials, or medical records. The questionnaire was translated from English to Bengali, adapted and validated per standard procedure. Data collection procedures were standardized across study sites through coordinated training of field staff conducted by epidemiologists, study physicians, and WHO staff members.

Blood pressure was measured by a trained field interviewer using the LifeSource UA-767+ blood pressure monitor, as recommended by the WHO, and appropriately sized arm cuffs. Blood pressure measurements were consistently taken on each participant's right arm at the level of the heart and elbow-assisted, while the participant was in a seated position. The initial measurement was performed after five minutes of rest. After two minutes, the second measurement was taken. The mean of these two blood pressure readings was utilized as the final blood pressure for each participant.

Following data collection, participants scheduled a visit with the study research physician within the following five days. The research physician assessed the participant's medical history, either through self-report or using medical records when possible. Study physicians examined each participant to confirm the results of data collection through classical symptom assessment. When necessary, the participant was also evaluated by the divisional investigator for a second opinion of relevant diagnoses.

Patient and public involvement

For peer review only - http://bmjopen.bmj.com/site/about/guidelines.xhtml

For peer review only

There was no patient or public involvement in the implementation of this study or interpretation of analytic results.

Outcome definitions

Our primary outcome of interest was the prevalence of hyperglycemia. We utilized the American Diabetes Association (ADA) guidelines to define a diagnosis of hyperglycemia using random or casual plasma glucose test and symptom review by the study physician [21]. An individual was considered to have hyperglycemia if the plasma glucose level was 11.1 mmol/L or higher (i.e. in the diabetic range) and/or if they self-reported to take diabetes medication. Our secondary outcome of interest was the prevalence of self-reported type-2 diabetes mellitus. An individual was categorized as diabetic if they self-reported to have been previously diagnosed with diabetes by a health care provider.

Covariates

The following variables were assessed as covariates for analysis: area of residence, sex, age, education, occupation, wealth index, body mass index (BMI), BP, and waist circumference. Education was categorized into four groups: no education, primary education (completed grade ≤5), secondary education (completed ≤ grade 10), and above secondary education (completed ≥ grade 12). Each participant's occupation was categorized into five groups, including: professional employment (field staff, police officer, guard, doctor, engineer, professional, businessman, desk job), unemployed or retired, industrial worker or day laborer, housewife, and other (shop keeper, weaver, driver, student, beggar, cook, carpenter, tailor, migrant workers and fishermen).

Data on physical activity was collected based on self-report. First, respondents were asked the number of days they engaged in vigorous, moderate, or light physical activity throughout a typical week. The following definitions were used to define (1) vigorous, (2)

For peer review only - http://bmjopen.bmj.com/site/about/guidelines.xhtml

moderate, and (3) light physical activity, respectively: (1) vigorous activity was defined as any activity that caused a large increase in breathing or heart rate, if continued for at least 10 minutes (e.g. running, carrying heavy loads, digging or construction work); (2) moderate activity was defined as any activity that caused a small increase in breathing or heart rate, if continued for at least 10 minutes (brisk walking or carrying light loads); and (3) light physical activity was defined as activities such as office work. Next, we asked participants to estimate how many minutes per day they engaged in the activity. MET-minute was calculated using the STEPS protocol[22] as follows: one minute of light activity was equivalent to 1 MET-minute; one minute in moderate-intensity activities was equivalent to 4 MET-minutes, and one minute of vigorous-intensity was equivalent to 8 MET-minutes. Physical activity was then categorized based on total MET-minutes per week. Participants who spent 3000 or more MET-minutes per week were categorized in the vigorous physical activity group, 600-3000 MET-minutes were categorized as moderate physical activity, and <600 MET-minutes were categorized as low physical activity.

The wealth index was constructed using principal component analysis. Asset information collected covered information on household ownership of nineteen items, including electricity, flush toilet, land telephone, cell phone, television, radio, refrigerator, car, motorcycle, washing machine, bicycle, sewing machine, wardrobe, table, bed or cot, chair or bench, watch or clock. Additionally, we assessed the main type of material used to build each participant's home (i.e. cement, tin, bamboo, or thatched straw). Each asset was assigned a weight (factor score) generated through principal components analysis, and the resulting asset scores were standardized to a normal distribution with a mean of zero and standard deviation of one. Each household was then assigned a score for each asset, and the scores were summed up; individuals were ranked according to the total score of the household in which they resided. The sample was then divided into quartiles from quartile one (lowest) to quartile four (highest). Using height (centimeters) and weight (kilograms) measurements, we calculated BMI (height/weight^2). BMI was categorized in the following groups: underweight (≤ 18.5), normal

For peer review only

(≤25), overweight (25.1-30) and obese (>30). Waist circumference was measured in centimeters (cm). Participants were categorized as abdominally obese if waist circumference was 90 cm and above for men, or 80 cm and above for women. Prehypertension was defined as SBP ≥120 mmHg but < 140 mmHg and/or DBP ≥ 80 mmHg but <90 mmHg and not taking antihypertensive medication at the time of the survey. We utilized the WHO's guidelines for cut-off points to define hypertension[23]. An individual was considered to have hypertension if systolic blood pressure (SBP) was ≥140 mmHg (millimeters of mercury) and/or, diastolic blood pressure (DBP) ≥90 mmHg, and/or taking antihypertensive medication based on self-report.

Age-Standardized Prevalence Estimates

To facilitate comparison of overall hyperglycemia among Bangladeshi adults across global populations with different age compositions we calculated age-standardized prevalence estimates with 95% confidence intervals (CIs) using the WHO's World Standard Population [24]. The World Standards database (WHO 2000-2025) provided population estimates for 18 and 19 age groups, as well as single year ages. To derive single ages from the 5-year age group proportions publically available, we used the Beers "Ordinary" Formula[25]. The following formula was utilized for standardization:

$\sum pi*w_i / \sum w_i$, where p = observed prevalence and w = world population weight.

Data Analysis

Sociodemographic variables were presented with mean and standard deviation (SD) for continuous variables, and using proportions for categorical variables. For bivariate analyses, study participants were divided by sex and into four age groups (18-29, 30-44, 45-54 and ≥55 years). We calculated the prevalence of our primary outcome by key demographic variables and calculated 95% CIs using the binomial exact method.

For peer review only - http://bmjopen.bmj.com/site/about/guidelines.xhtml

To estimate determinants of hyperglycemia in the diabetic range, we computed prevalence ratios with Poisson regression using robust estimation of standard errors[26-28]. Potential variables for inclusion in the model were assessed using prior published literature and bivariate Poisson regression analysis; an arbitrary p-value of <0.10 was used as criteria to include the variable in the multivariable Poisson regression model to control for confounding effects. For multivariable Poisson regression models, adjusted prevalence ratios (aPR), and 95% CIs for each independent variable were calculated. Additionally, *<0.05* was used as the level of significance. Multivariable Poisson regression models were generated separately for urban and rural participants to account for possible effect measure modification. Collinearity was assessed using the variance inflation factor to ensure a strong linear relationship among independent variables included in the model was not present. All statistical procedures were performed using Stata/SE 15.0 (StataCorp LP, Texas, USA) software package.

Results

Background Characteristics

Of the 2000 adults approached, 1843 agreed to participate in our study leading to a response rate of 92.1%. Twenty-four female participants were pregnant and were dropped from subsequent analyses to ensure those with gestational diabetes were not included. Additionally, no participants reported having been previously diagnosed with type-1 diabetes. There were 892 (49.0%) male and 927 (50.9%) female respondents (Table 1). The age of our participants ranged from 18 to 90 years. The mean age and education level of participants were 40.5 (SD = 14.7) years and 5.7 (SD = 5.1) years, respectively. The majority of the study population was married (88.0%) and employed as either an industrial worker/day laborer (26.5%) or housewife (39.9%). Almost half of participants never used some form of tobacco. The majority of participants engaged in vigorous physical activity over an average week (69.3%). The mean BMI was 22.1 (SD = 4.1) and the mean waist circumference was 78.4 cm (SD = 11.6). Overall,

For peer review only - http://bmjopen.bmj.com/site/about/guidelines.xhtml

the mean systolic blood pressure was 116.1 mmHg (SD = 17.1) and diastolic blood pressure was 76.1 mmHg (SD = 10.5). The mean blood glucose level was 6.4 mmol/L (SD = 2.4). Figure 1 presents the distribution of blood glucose levels by various demographic factors.

Prevalence and Risk Factors for Hyperglycemia

The prevalence of hyperglycemia was 5.5% (95% CI: 4.5-6.6). This prevalence was significantly higher among urban participants (9.8%, 95%CI: 7.7-12.2) than rural participants (2.8%, 95% CI: 1.9-3.9) (Table 2) and increased as age increased (Figure 2). The highest prevalence of hyperglycemia was observed among those aged ≥55 years, at 8.2% (95% CI: 4.6-13.1) among men and 9.4% (95% CI: 5.4-14.8) among women. The age-standardized prevalence of hyperglycemia was 5.6% (95% CI: 4.6-6.8). The age-standardized prevalence of hyperglycemia among urban and rural residents was 10.5% (95% CI: 9.2-12.1) and 2.8% (95% CI: 2.1-3.7), respectively. Among men and women, the age-standardized prevalence of hyperglycemia was 4.9% (95% CI: 3.9-6.0) and 6.0% (95% CI: 4.9-7.2), respectively.

Self-Reported Diabetes Mellitus and Hyperglycemia

Ninety-five participants (5.2%) self-reported to have been previously diagnosed with type-2 diabetes by a health care provider. However, 25 of those participants did not meet our criteria of diagnosis of hyperglycemia as they did not take medication to control their diabetes and their plasma glucose was below 11.1 mmol/L. Therefore, 69.3% of those with hyperglycemia, per the study definition, were previously diagnosed with diabetes by a health care provider.

The proportion of men and women who were previously diagnosed with diabetes by a health care provider based on self-report was higher among urban residents (men: 8.4%; women: 9,1%) than among rural residents (men: 2.9%; women: 3.0%) (Figure 3a). However, overall, the large majority (81.8%) reported they did not know if they had been previously

For peer review only - http://bmjopen.bmj.com/site/about/guidelines.xhtml

diagnosed with diabetes; this proportion was higher among rural residents (87.8%) than urban residents (72.2%).

Among participants previously diagnosed with type-2 diabetes based on self-report, 72.6% reported taking medication to control their diabetes (Table 1). Urban women more frequently (96.7%) self-reported to take diabetes medication than urban men (62.1%) (Figure 3b). We were able to confirm 100% of participant's self-reported diabetes treatment history by checking prescriptions or medicine strips/vials.

Among participants who were categorized as hyperglycemic during study measurement, over one-third (37.9%) of urban men self-reported to have diabetes, however, they did not take any medication to control their diabetes. Among rural participants, the proportion of women who did not take medication to control their self-reported diabetes was higher (52.9%) than men (31.3%). Although three-quarters of self-reported diabetic participants reported taking medication to control their diabetes, 31% continued to have high blood sugar levels indicating uncontrolled diabetes at study measurement (Figure 4).

Determinants of Hyperglycemia

Table 2 presents the results of multivariable Poisson regression with robust variance analyses to identify determinants of hyperglycemia. Among urban participants, those of older age, lowest wealth quartile, hypertension, low physical activity, and with abdominal obesity based on waist circumference, were more likely to have hyperglycemia. The prevalence of hyperglycemia was significantly highest among those aged ≥55 years (aPR 3.92, 95% CI: 1.48-10.39) compared to individuals aged 18-29 years of age. When compared to those in the 4th (highest) wealth quartile, urban residents in the first (lowest) wealth quartile had 3.18 times the prevalence of hyperglycemia. For urban individuals with hypertension, the prevalence of hyperglycemia was 2.65 (95% CI:1.30-5.38) times that of individuals without hypertension. The prevalence of hyperglycemia among those with low physical activity was 3.01 (95% CI: 1.42-

For peer review only - http://bmjopen.bmj.com/site/about/guidelines.xhtml

6.38) times that of urban participants with vigorous physical activity. Abdominal obesity also significantly increased the prevalence of hyperglycemia among urban participants (aOR: 2.54, 95% CI: 1.35-4.77). Among rural participants, the only observed determinants of hyperglycemia were hypertension (aPR: 5.39, 95% CI: 1.94-14.96) and abdominal obesity (aPR: 2.95, 95% CI: 1.32-6.58).

Discussion

Using data from this nationally representative sample, we estimate that about one in twenty Bangladeshi adults aged ≥18 years have hyperglycemia. The prevalence of hyperglycemia was higher among urban residents (9.8%) than rural residents (2.8%). Risk factors of hyperglycemia included older with age, urban residence, abdominal obesity, low physical activity, and hypertension. As diabetes is characterized by hyperglycemia, targeting high-risk groups identified in this analysis could be prioritized for effective diabetes preventive programs in Bangladesh. Bangladesh has adopted the goals and targets set forth by the WHO's Global Monitoring Framework for the Prevention and Control of NCDs for the year 2025[11]. One of these targets is to ensure there is a 0% increase in the age-standardized prevalence of hyperglycemia among adults aged ≥18 years by the year 2025. To our knowledge, the present study is the first to report national estimates on the prevalence of hyperglycemia starting at age 18 years in both urban and rural areas of Bangladesh. Data gathered from this national-level study are critical towards the measurement of progress towards the nine global targets for Bangladesh for 2025.

Globally, the number of adults living with diabetes has risen from 108 million in 1980 to 422 million in 2013, and low- and middle-income countries (LMICs) have seen the most rapid rise in diabetes prevalence[3]. Several lifestyle factors have been attributed to the increase in prevalence across LMICs including, globalization of food production, extensive marketing of low-cost and energy-dense foods, increased sedentary behavior and rapid urbanization[29]. In

For peer review only - http://bmjopen.bmj.com/site/about/guidelines.xhtml

recent decades, the increase in the prevalence of diabetes in South Asia has been greater than that seen in high-income countries[4]. The prevalence of diabetes in adults across countries in South Asia is similar, excluding Nepal which has a low prevalence in comparison to neighboring countries (8.8% in India, 8.6% in Sri Lanka, 6.9% in Bangladesh, 7.9% in Bhutan, 6.9% in Pakistan, and 4.0% in Nepal)[30]. In our study, we observed a prevalence of 5.6% hyperglycemia in the diabetic range among adults aged 18 years and above. This prevalence is lower than previous studies conducted in Bangladesh and neighboring countries. In fact, a scoping review estimated the pooled prevalence of type-2 diabetes to be 7.4%[7]. Our estimated prevalence was lower due to a younger study population (18 years and above compared to the WHO estimate among adults aged 30 years and above), and a higher percentage of participants from rural areas (61.1%, as is representative of Bangladesh). Indeed, when we restrict our analytic sample to 30 years and above, the prevalence of hyperglycemia is 6.7%, which is similar to the 2017 WHO estimate of diabetes (6.9%). Significant heterogeneity in diabetes and its determinants may exist within one country due to variations in the level of urbanization by state, ethnic phenotypes, and socioeconomic status of specific sub-populations[31].

Significant determinants of raised blood glucose in both urban and rural areas of our assessment included hypertension, low physical activity, and abdominal obesity. Interestingly, there was no association of diabetes identified for increasing body mass index (BMI). This indicates that abdominal obesity may be a more significant factor to consider than BMI. Prior studies conducted in Bangladesh have also identified a positive association of central (or abdominal) obesity with diabetes[32]. Interestingly, our assessment found a decrease in odds of diabetes with increasing wealth quartile. Prior studies have conflicting findings on the risk of diabetes and other NCDs, among the wealthy based on demographic features such as the area of residence. One prior study conducted in Bangladesh found that people from the highest wealth quintile are significantly more likely to have diabetes than people from the lowest wealth quintile[33]. However, another found a high burden of selected NCDs, including diabetes, among

For peer review only - http://bmjopen.bmj.com/site/about/guidelines.xhtml

the lowest wealth quintile populations in rural areas and wealthy populations in urban areas[34]. Further study is warranted to assess the reliability of wealth indices as a measurement of socioeconomic status and wealth among Bangladeshi adults.

In our study, a high proportion (~70%) of those with hyperglycemia self-reported to have been previously diagnosed with diabetes and therefore, aware of their condition. Additionally, 72% reported taking medication to control their diabetes. However, we found that almost one-third of those who self-reported to take medication for their diabetes continued to have hyperglycemia. Efforts should be made to ensure diabetics in Bangladesh are treated for their condition and secondary prevention of complications of diabetes, such as diabetic retinopathy. This is of particular concern in developing countries where resources are limited and cost-effective solutions for chronic disease treatment should be prioritized. A recently published study found that healthcare expenditure in persons with diabetes in Bangladesh is six times higher than in persons without diabetes [14]. Prevention and management of diabetes are likely to be a cost-saving approach for Bangladesh through the utilization of community health workers adequately trained to effectively screen for, and identify, people with diabetes [35].

This study has several strengths. Data collected for our study was of a nationally representative sample indicating our results are generalizable to the population of Bangladeshi adults aged 18 years and above. Additionally, due to our large sample size, we were able to conduct precise subgroup analyses. However, several limitations should also be considered when interpreting the results of this analysis. We assessed blood glucose levels using random capillary blood samples, however, we did not assess each participant's history of classical symptoms of hyperglycemia, which is necessary to diagnose diabetes according to the ADA guidelines[21]. Additionally, we were unable to measure known determinants of type-2 diabetes factors such as diet or family history of diabetes. Further, we did not collect blood glucose samples in fasting or two-hour post-prandial status to also assess prediabetes. Future studies should consider the addition of glycosylated hemoglobin measurement when assessing the

For peer review only - http://bmjopen.bmj.com/site/about/guidelines.xhtml

prevalence of diabetes as this method could provide a more long-term and stable diagnosis of diabetes mellitus. Finally, due to the cross-sectional nature of this study, we were unable to define temporality of certain determinants of hyperglycemia identified and therefore, unable to assess causality.

Conclusion

Data from this nationally-representative sample of Bangladeshi adults aged 18 years and above will be critical to informing the progress of NCD control in Bangladesh per the WHO's Global Monitoring Framework and goals for 2025. We found that about one in twenty Bangladeshi adults aged ≥18 years have hyperglycemia. Among urban residents, we found that about one in ten Bangladeshi adults aged ≥18 years have hyperglycemia. Bangladeshi adults with hypertension and abdominal obesity are high-risk groups for the development of diabetes and should be targeted for routine screening for diabetes. Preventive methods such as lifestyle changes and medication should be recommended by primary care providers in Bangladesh to avoid the future development of CVDs among this group. In order to control the prevalence of hyperglycemia in the diabetic range, and reduce the burden of diabetes or associated risk factors, national initiatives such as training community health workers to deliver primary care and implementing universal health coverage should be implemented to curb the spread of NCDs in Bangladesh.

For peer review only

For peer review only - http://bmjopen.bmj.com/site/about/guidelines.xhtml

Figures

Figure 1: Urban and Rural differences in the distribution of blood glucose levels based on random capillary blood measurement among (A) All Participants, and (B) Men and Women (n = 1819)

Figure 2: Prevalence of hyperglycemia among Bangladeshi adults aged 18 years and above by sex and age group, 2015 (n = 1819)

Figure 3: Bangladeshi adults aged 18 years and above with (A) self-reported diabetes and (B) self-reported diabetics on diabetes medications, 2015

Figure 4: Self-reported diabetics aged 18 years or older who take diabetes medication with hyperglycemia on study measurement (≥11.0mmol/L)

For peer review only

For peer review only - http://bmjopen.bmj.com/site/about/guidelines.xhtml

Abbreviations

WHO: World Health Organization

ADA: American Diabetes Association

aPR: Adjusted prevalence ratio

BBS: Bangladesh Bureau of Statistics

BMI: Body mass index

BP: Blood pressure

BSMMU: Bangabandhu Sheikh Mujib Medical University

CI: Confidence interval

CVD: Cardiovascular disease

MET: Metabolic equivalent

mmHg: Millimeter of mercury

mmol/L: Millimoles per liter

NCD: Non-communicable disease

SBP: Systolic blood pressure

DBP: Diastolic blood pressure

PSU: Primary sampling unit

SD: Standard deviation

STEPS: STEPwise approach to Surveillance

For peer review only

Footnotes

Ethics approval and consent to participate: Ethical guidelines as outlined by the Declaration of Helsinki were followed throughout the study. Ethical clearance was obtained from the Institutional Review Board of Bangabandhu Sheikh Mujib Medical University (BSMMU) (Protocol Number: 1100). We obtained permission from the relevant administrative units of the surveyed districts. Orientations with community leaders (elected representatives of the local government offices) were conducted prior to data collection for community engagement in the study's implementation process. Written (or thumb impression if unable to write) consent was obtained from the respondents in Bangla as per BSMMU Institutional Review Board (IRB) guidelines.

Consent to publish: All authors consent to the publication of this manuscript.

Availability of data and materials: The de-identified participant data used and/or analyzed during the current study are available from the corresponding author on reasonable request. Please contact M. Mostafa Zaman at zamanm@who.int for further information and guidelines.

Competing Interests: The authors declare no competing interests. The authors alone are responsible for views expressed in this article and they do not necessarily represent the views, decisions or policies of the institutions with which they are affiliated.

Funding: The study was conducted with the technical and financial assistance of the World Health Organization Country Office for Bangladesh.

Authors contributions: JYI: conceptualized the manuscript, analyzed data, interpreted results critically, and drafted the manuscript. MMZ: designed the study, interpreted results critically, guided manuscript writing, and critically reviewed it. MRB, SAH, SA, ZAQ: trained the field team, implemented the survey, processed and analyzed data, and reviewed the manuscript.

Acknowledgments: The authors thank Mr. Hassanuzzaman Khan for his efforts on data management.

For peer review only

For peer review only - http://bmjopen.bmj.com/site/about/guidelines.xhtml

References

1. Disease GBD, Injury I, Prevalence C. Global, regional, and national incidence, prevalence, and years lived with disability for 354 diseases and injuries for 195 countries and territories, 1990-2017: a systematic analysis for the Global Burden of Disease Study 2017. *Lancet (London, England)* 2018;392(10159):1789-858. doi: 10.1016/S0140-6736(18)32279-7
2. Ogurtsova K, da Rocha Fernandes JD, Huang Y, et al. IDF Diabetes Atlas: Global estimates for the prevalence of diabetes for 2015 and 2040. *Diabetes research and clinical practice* 2017;128:40-50. doi: 10.1016/j.diabres.2017.03.024
3. Global report on diabetes. Geneva, Switzerland: World Health Organization 2016.
4. Jayawardena R, Ranasinghe P, Byrne NM, et al. Prevalence and trends of the diabetes epidemic in South Asia: a systematic review and meta-analysis. *BMC public health* 2012;12:380. doi: 10.1186/1471-2458-12-380
5. IDF diabetes atlas sixth edition Brussels, Belgium Interational Diabetes Federation, 2013.
6. Saquib N, Saquib J, Ahmed T, et al. Cardiovascular diseases and type 2 diabetes in Bangladesh: a systematic review and meta-analysis of studies between 1995 and 2010. *BMC public health* 2012;12:434. doi: 10.1186/1471-2458-12-434
7. Biswas T, Islam A, Rawal LB, et al. Increasing prevalence of diabetes in Bangladesh: a scoping review. *Public health* 2016;138:4-11. doi: 10.1016/j.puhe.2016.03.025
8. Rahman MS, Akter S, Abe SK, et al. Awareness, treatment, and control of diabetes in Bangladesh: a nationwide population-based study. *PloS one* 2015;10(2):e0118365. doi: 10.1371/journal.pone.0118365
9. Akter S, Rahman MM, Abe SK, et al. Prevalence of diabetes and prediabetes and their risk factors among Bangladeshi adults: a nationwide survey. *Bulletin of the World Health Organization* 2014;92(3):204-13, 13A. doi: 10.2471/BLT.13.128371

For peer review only - http://bmjopen.bmj.com/site/about/guidelines.xhtml

10. Zaman MM, Choudhury SR, Ahmed J, et al. Blood glucose and cholesterol levels in adult population of Bangladesh: Results from STEPS 2006 survey. *Indian heart journal* 2016;68(1):52-6. doi: 10.1016/j.ihj.2015.06.031

11. NCD Global Monitoring Framework: World Health Organization; 2017 [Available from: http://www.who.int/nmh/global_monitoring_framework/en/ accessed 09/29 2017.

12. Global action plan for the prevention and control of noncommunicable diseases 2013-2020. Geneva, Switzerland: World Health Organization, 2013.

13. Siddique MKB, Islam SMS, Banik PC, et al. Diabetes knowledge and utilization of healthcare services among patients with type 2 diabetes mellitus in Dhaka, Bangladesh. *BMC health services research* 2017;17(1):586. doi: 10.1186/s12913-017-2542-3

14. Shariful Islam SM, Lechner A, Ferrari U, et al. Healthcare use and expenditure for diabetes in Bangladesh. *BMJ Glob Health* 2017;2(1):e000033. doi: 10.1136/bmjgh-2016-000033

15. Haghparast-Bidgoli H, Shaha SK, Kuddus A, et al. Protocol of economic evaluation and equity impact analysis of mHealth and community groups for prevention and control of diabetes in rural Bangladesh in a three-arm cluster randomised controlled trial. *BMJ open* 2018;8(8):e022035. doi: 10.1136/bmjopen-2018-022035

16. Islam JY, Zaman MM, Haq SA, et al. Epidemiology of hypertension among Bangladeshi adults using the 2017 ACC/AHA Hypertension Clinical Practice Guidelines and Joint National Committee 7 Guidelines. *Journal of human hypertension* 2018;32(10):668-80. doi: 10.1038/s41371-018-0087-5

17. Chronic Disease and Health Promotion: STEP wise approach to surveillance (STEPS). Geneva: World Health Organization.

18. Population and Housing Census 2001, updated in 2009. Dhaka, Bangladesh: Bangladesh Bureau of Statistics 2009.

For peer review only - http://bmjopen.bmj.com/site/about/guidelines.xhtml

19. Goycochea-Robles MV, Sanin LH, Moreno-Montoya J, et al. Validity of the COPCORD core questionnaire as a classification tool for rheumatic diseases. *J Rheumatol Suppl* 2011;86:31-5. doi: 10.3899/jrheum.100955

20. Guidelines for Care of Type 2 Diabetes Mellitus in Bangaldesh. Dhaka, Bangladesh: BIRDEM Clinical Research Group, 2003.

21. American Diabetes A. 2. Classification and Diagnosis of Diabetes. *Diabetes care* 2017;40(Suppl 1):S11-S24. doi: 10.2337/dc17-S005

22. Moniruzzaman M, Mostafa Zaman M, Islam MS, et al. Physical activity levels in Bangladeshi adults: results from STEPS survey 2010. *Public health* 2016;137:131-8. doi: 10.1016/j.puhe.2016.02.028

23. A global brief on Hypertension: Silent Killer, global public health crisis. Geneva, Switzerland: World Health Organization, 2013.

24. Ahmad OB, Boschi-Pinto C, Lopez AD, et al. Age Standardization of Rates: A New WHO Standard. GPE Discussion Paper Series: No31 Geneva, Switzerland: World Health Organization, 2001.

25. World (WHO 2000-2025) Standard Rockville, MD: National Cancer Institute: Surveillance, Epidemiology, and End Results Program; 2017 [Available from: https://seer.cancer.gov/stdpopulations/world.who.html accessed December 17 2017.

26. Coutinho LM, Scazufca M, Menezes PR. Methods for estimating prevalence ratios in cross-sectional studies. *Revista de saude publica* 2008;42(6):992-8.

27. Behrens T, Taeger D, Wellmann J, et al. Different methods to calculate effect estimates in cross-sectional studies. A comparison between prevalence odds ratio and prevalence ratio. *Methods Inf Med* 2004;43(5):505-9.

28. Barros AJ, Hirakata VN. Alternatives for logistic regression in cross-sectional studies: an empirical comparison of models that directly estimate the prevalence ratio. *BMC medical research methodology* 2003;3:21. doi: 10.1186/1471-2288-3-21

For peer review only - http://bmjopen.bmj.com/site/about/guidelines.xhtml

29. Singh PN, Arthur KN, Orlich MJ, et al. Global epidemiology of obesity, vegetarian dietary patterns, and noncommunicable disease in Asian Indians. *The American journal of clinical nutrition* 2014;100 Suppl 1:359S-64S. doi: 10.3945/ajcn.113.071571

30. Hills AP, Arena R, Khunti K, et al. Epidemiology and determinants of type 2 diabetes in south Asia. *Lancet Diabetes Endocrinol* 2018;6(12):966-78. doi: 10.1016/S2213-8587(18)30204-3

31. Chan JC, Malik V, Jia W, et al. Diabetes in Asia: epidemiology, risk factors, and pathophysiology. *Jama* 2009;301(20):2129-40. doi: 10.1001/jama.2009.726

32. Alam DS, Talukder SH, Chowdhury MA, et al. Overweight and abdominal obesity as determinants of undiagnosed diabetes and pre-diabetes in Bangladesh. *BMC Obes* 2016;3:19. doi: 10.1186/s40608-016-0099-z

33. Tareque MI, Koshio A, Tiedt AD, et al. Are the rates of hypertension and diabetes higher in people from lower socioeconomic status in Bangladesh? Results from a nationally representative survey. *PloS one* 2015;10(5):e0127954. doi: 10.1371/journal.pone.0127954

34. Biswas T, Islam MS, Linton N, et al. Socio-Economic Inequality of Chronic Non-Communicable Diseases in Bangladesh. *PloS one* 2016;11(11):e0167140. doi: 10.1371/journal.pone.0167140

35. Gaziano TA, Abrahams-Gessel S, Denman CA, et al. An assessment of community health workers' ability to screen for cardiovascular disease risk with a simple, non-invasive risk assessment instrument in Bangladesh, Guatemala, Mexico, and South Africa: an observational study. *The Lancet Global health* 2015;3(9):e556-63. doi: 10.1016/S2214-109X(15)00143-6

For peer review only

For peer review only - http://bmjopen.bmj.com/site/about/guidelines.xhtml

Table 1: Background Characteristics of Bangladeshi adult participants, 2015 (n = 1819)

	Total (n = 1819)			Urban (n = 708)			Rural (n = 1111)		
Characteristic	Mean (SD)	n	%	Mean (SD)	n	%	Mean (SD)	n	%
Sex									
Male		892	49.0		345	48.7		547	49.2
Female		927	50.9		363	51.3		564	50.8
Age(years)	40.5 (14.7)			39.1 (13.9)			41.4 (15.1)		
Education (Years) [a]	5 (0 - 9)			8 (3-12)			4 (0-8)		
Marital Status									
Never Married		110	6.1		54	7.6		56	4.9
Married		1601	88.0		619	87.4		982	88.4
Separated/Divorced/Widowed		108	5.9		35	4.9		73	6.5
Occupation									
Professional employment [b]		279	15.2		189	26.7		90	8.1
Unemployed/retired		98	5.3		43	6.1		55	4.9
Industrial worker/Day Laborer		483	26.6		120	16.9		363	32.7
Housewife		726	39.9		247	34.9		479	43.2
Other [c]		232	12.8		109	15.4		123	11.1
Wealth Index [d]									
1st Wealth Quartile		407	22.4		110	15.5		297	26.7
2nd Wealth Quartile		533	29.3		171	24.2		362	32.6
3rd Wealth Quartile		429	23.6		179	25.3		250	22.5
4th Wealth Quartile		450	24.7		248	35.0		202	18.2
Tobacco Use [e]									
Never		859	47.2		389	54.9		470	42.3
Current Use		821	45.1		268	37.9		553	49.8
Past Use		139	7.6		51	7.2		88	7.9
Smoking Tobacco Use [f]									
Every day / Occasionally		494	27.2		169	23.9		325	29.3
Past Use		104	5.7		38	5.4		66	5.9
Never		1221	67.1		501	70.8		720	64.8
Smokeless Tobacco Use [g]									
Every day / Occasionally		529	29.1		150	21.2		379	34.1
Past Use		51	2.8		21	2.9		30	2.7
Never		1239	68.1		537	75.9		702	63.2
Physical Activity [h]									
Vigorous		1268	69.7		445	62.9		823	74.1
Moderate		464	25.5		234	33.1		230	20.7
Low		87	4.7		29	4.1		58	5.2

For peer review only

For peer review only - http://bmjopen.bmj.com/site/about/guidelines.xhtml

Body Mass Index [i]	22.1 (4.1)		23.3 (4.5)		21.3 (3.7)	
Waist Circumference (cm)	78.4 (11.6)		81.8 (12.6)		76.2 (10.3)	
Blood Pressure						
Systolic Blood Pressure (mmHg)	116.1 (17.1)		117.9 (16.6)		115.0 (17.3)	
Diastolic Blood Pressure (mmHg)	76.1 (10.5)		77.9 (10.9)		74.9 (10.1)	
Blood Glucose Level (mmol/l)	6.4 (2.4)		6.6 (2.9)		6.3 (2.1)	
Self-reported diabetes medication history [j]	69	72.6	50	80.6	19	57.6

Abbreviations: SD, standard deviation

[a] Calculated median and interquartile range for education as the data are skewed

[b] Professional occupation includes: Field staff, police officer, guard, doctor, engineer, professional, business man, desk job

[c] Other occupation includes: Shop keeper, weavers, driver, student, beggar, cook, carpenter, tailor, migrant workers and fishermen

[d] Wealth index was calculated using principal component analysis using data collected on household ownership of the following items: electricity, flushable toilet, land phone, cell phone, television, radio, refrigerator, private car, motor cycle, washing machine, bicycle, sewing machine, almirah/wardrobe, table, bed, chair/bench, watch/clock, as well as, type of main material used to build their homes roof, walls and floor

[e] Includes both smokeless tobacco and smoke tobacco

[f] Smoking tobacco use includes cigarettes, biri, hookah, etc.

[g] Smokeless tobacco use includes jarda, sada pata, pan mashala with tobacco leaf, gul etc.

[h] Measured in MET-minutes; 1 MET stands for the amount of oxygen you consume and the number of calories you burn at rest.

[i] Body mass index (BMI) calculated by weight in kilogram divided by height in meter squared

[j] Percentage reported out of participants who self-reported to have diabetes (Total n = 95; Urban n = 62; Rural n = 33)

For peer review only

Table 2: Determinants of hyperglycemia among Bangladeshi adults, 2015 (n = 1819)

	Total (n = 1819)		Urban (n = 708)		Rural (n = 1111)	
	Diabetes [a] Prevalence %	Adjusted PR [b] (95% CI)	Diabetes [a] Prevalence %	Adjusted OR [b] (95% CI)	Diabetes [a] Prevalence %	Adjusted OR [b] (95% CI)
**<u>Characteristic</u>**						
Area						
Urban	9.8	Ref.	-	-	-	-
Rural	2.8	0.44 (0.28 – 0.68)	-	-	-	-
Sex						
Male	4.9	Ref.	7.5	Ref.	3.3	Ref.
Female	5.9	1.05 (0.71 – 1.54)	11.9	1.26 (0.80 – 1.99)	2.2	0.61 (0.29 – 1.28)
Age (years)						
18 - 29	2.5	Ref.	2.5	Ref.	2.5	Ref.
30 - 44	4.9	1.48 (0.78 – 2.79)	8.4	2.55 (1.01 – 6.41)	2.5	0.76 (0.30 – 1.93)
45 - 54	7.4	2.18 (1.10– 4.31)	15.5	4.38 (1.62 – 11.59)	2.8	0.67 (0.25 – 1.83)
≥ 55	8.8	1.92 (0.95 – 3.86)	19.3	3.92 (1.48 – 10.39)	3.4	0.53 (0.17 – 1.67)
Educational Status						
No Education	3.4	Ref.	7.0	Ref.	2.1	Ref.
Primary Education	3.7	1.00 (0.53 – 1.86)	7.6	1.00 (0.44 – 2.30)	2.1	0.84 (0.30 – 2.30)
Secondary Education	6.9	1.67 (0.95 – 2.93)	12.1	1.94 (0.95 – 3.97)	2.9	1.01 (0.40 – 2.54)
Above Secondary Education	9.8	1.48 (0.77 – 2.84)	10.4	1.54 (0.71 – 3.33)	8.1	2.24 (0.68 – 7.41)
Wealth Index [c]						
1st Wealth Quartile	7.7	2.58 (1.57 – 4.24)	18.9	3.18 (1.80 – 5.62)	3.6	1.37 (0.53 – 3.49)
2nd Wealth Quartile	4.3	1.23 (0.71 – 2.14)	9.4	1.50 (0.82 – 2.77)	1.9	0.70 (0.24 – 2.05)
3rd Wealth Quartile	3.7	0.86 (0.47 – 1.58)	6.0	0.96 (0.48 – 1.92)	2.0	0.55 (0.18 – 1.73)
4th Wealth Quartile	6.6	Ref.	8.8	Ref.	3.9	Ref.
Blood Pressure						
Normal Blood Pressure	1.9	Ref.	3.5	Ref.	1.1	Ref.
Pre-Hypertension [d]	5.7	1.74 (1.00 – 3.01)	8.3	1.37 (0.69 – 2.74)	3.8	2.32(0.91 – 5.92)
Hypertension [e]	18.9	3.57 (2.01 – 6.34)	27.4	2.65 (1.30 – 5.38)	8.8	5.39(1.94 –14.96)
Physical Activity						
Vigorous	3.9	Ref.	6.9	Ref.	2.3	Ref.
Moderate	8.2	1.18 (0.78 – 1.77)	13.8	1.22 (0.77 – 1.93)	2.1	0.65 (0.22 – 1.88)
Low	14.9	3.04 (1.69 – 5.47)	20.7	3.01 (1.42 – 6.38)	12.1	2.58 (0.95 – 7.04)

For peer review only - http://bmjopen.bmj.com/site/about/guidelines.xhtml

Body Mass Index [f]						
Underweight (<18.5)	1.1	0.37 (0.12 – 1.13)	1.8	0.38 (0.07 – 1.95)	0.7	0.42 (0.09 – 2.02)
Normal (18.5 - 25)	4.7	Ref.	8.2	Ref.	2.8	Ref.
Overweight (25.1 - 30)	10.4	1.06 (0.68 – 1.65)	14.0	1.05 (0.61 – 1.80)	6.2	1.22 (0.51 – 2.91)
Obese (>30)	20.9	1.49 (0.87 – 2.57)	26.5	1.46 (0.81 – 2.64)	5.6	0.95 (0.12 – 7.60)
Waist Circumference (cm)						
Normal [g]	2.3	Ref.	3.8	Ref.	1.6	Ref.
Abdominally Obese [h]	13.3	2.49 (1.53 – 4.07)	18.1	2.54 (1.35 – 4.77)	6.8	2.95 (1.32 – 6.58)

Abbreviations: PR = prevalence ratio, CI = confidence intervals, Ref = referent category

[a] Hyperglycemia was defined as a capillary blood glucose level greater than or equal to 11.1 mmol/L or self-reported diabetes medication use

[b] Model adjusted for all variables included in table: sex, age, education, wealth index, blood pressure, body mass index, self-reported physical activity, and waist circumference

[c] Wealth index was calculated using principal component analysis using data collected on household ownership of the following items: electricity, flushable toilet, land phone, cell phone, television, radio, refrigerator, private car, motor cycle, washing machine, bicycle, sewing machine, almirah/wardrobe, table, bed, chair/bench, watch/clock, as well as, type of main material used to build their homes roof, walls and floor

[d] Pre-Hypertension was defined as SBP ≥120 mmHg but < 140 mmHg and/or DBP ≥ 80 mmHg but <90 mmHg and not taking anti-hypertensive medication at the time of the survey

[e] Hypertension was defined as systolic blood pressure (SBP) was ≥140 mmHg (millimeters of mercury) and/or, diastolic blood pressure (DBP) ≥90 mmHg and/or taking any-hypertensive medication

[f] Body mass index (BMI) calculated by weight in kilogram divided by height in meter squared

[g] Defined as <90 cm M; <80 cm F

[h] Defined as ≥90 cm M; ≥80 cm F

For peer review only

For peer review only - http://bmjopen.bmj.com/site/about/guidelines.xhtml

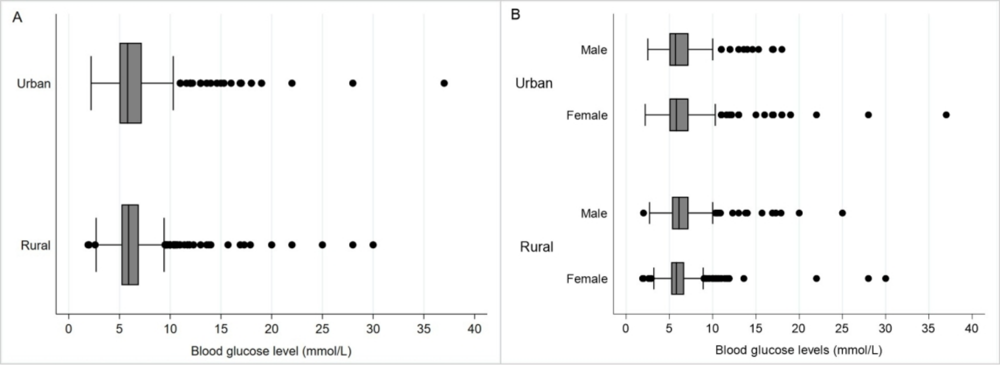

Figure 1: Urban and Rural differences in the distribution of blood glucose levels based on random capillary blood measurement among (A) All Participants, and (B) Men and Women (n = 1819)

327x119mm (150 x 150 DPI)

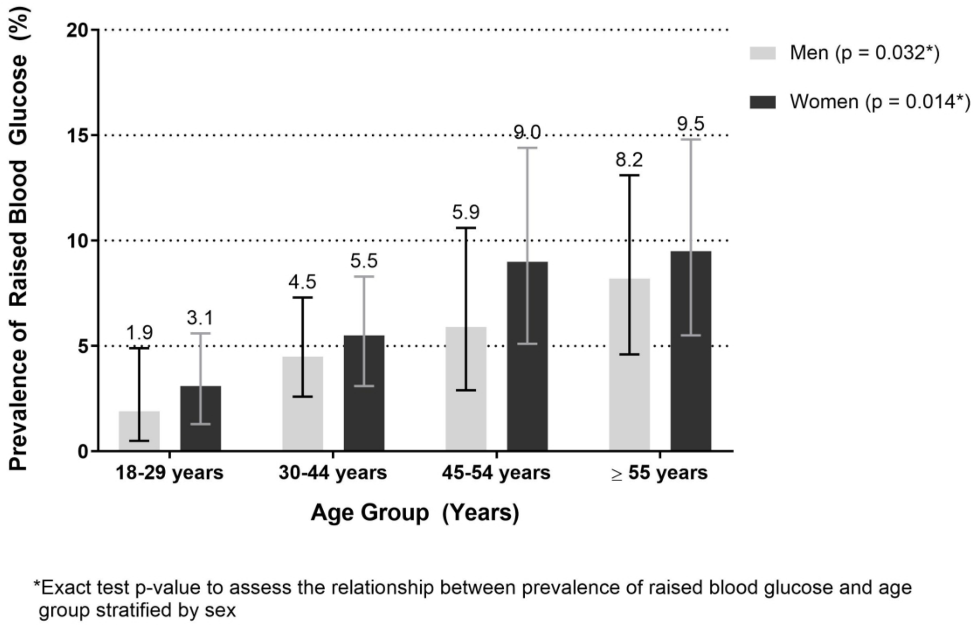

Figure 2: Prevalence of hyperglycemia among Bangladeshi adults aged 18 years and above by sex and age group, 2015 (n = 1819)

211x137mm (300 x 300 DPI)

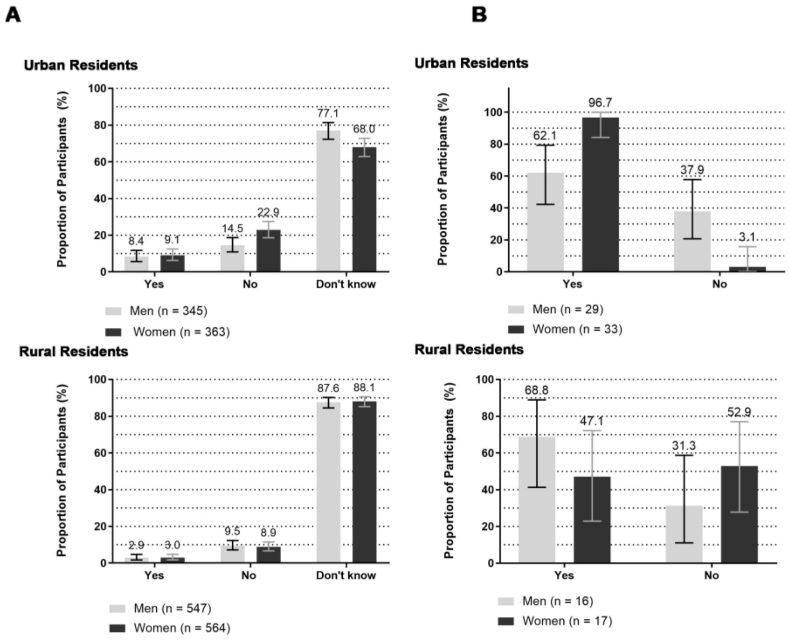

Figure 3: Bangladeshi adults aged 18 years and above with (A) self-reported diabetes and (B) self-reported diabetics on diabetes medications, 2015

284x190mm (300 x 300 DPI)

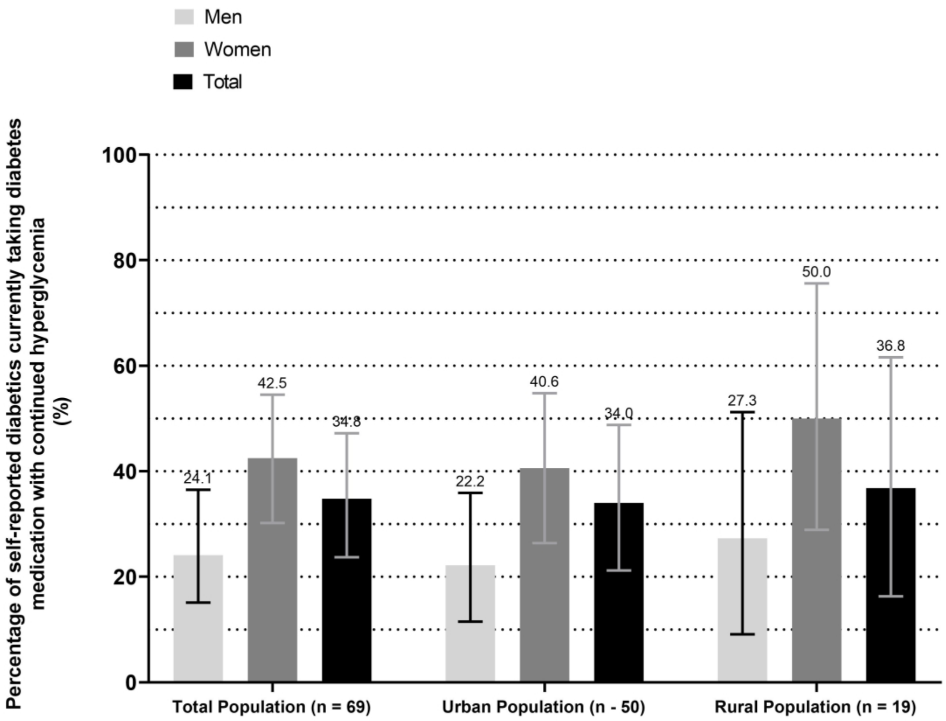

Figure 4: Self-reported diabetics aged 18 years or older who take diabetes medication with hyperglycemia on study measurement (≥11.0mmol/L)

284x210mm (300 x 300 DPI)

STROBE 2007 (v4) Statement—Checklist of items that should be included in reports of *cross-sectional studies*

Section/Topic	Item #	Recommendation	Reported on page #
**Title and abstract**	1	(*a*) Indicate the study's design with a commonly used term in the title or the abstract	2
		(*b*) Provide in the abstract an informative and balanced summary of what was done and what was found	2-3
**Introduction**			
Background/rationale	2	Explain the scientific background and rationale for the investigation being reported	5-6
Objectives	3	State specific objectives, including any prespecified hypotheses	6
**Methods**			
Study design	4	Present key elements of study design early in the paper	6
Setting	5	Describe the setting, locations, and relevant dates, including periods of recruitment, exposure, follow-up, and data collection	7
Participants	6	(*a*) Give the eligibility criteria, and the sources and methods of selection of participants	6
Variables	7	Clearly define all outcomes, exposures, predictors, potential confounders, and effect modifiers. Give diagnostic criteria, if applicable	8-9
Data sources/ measurement	8*	For each variable of interest, give sources of data and details of methods of assessment (measurement). Describe comparability of assessment methods if there is more than one group	7-10
Bias	9	Describe any efforts to address potential sources of bias	6-7 & 11
Study size	10	Explain how the study size was arrived at	7
Quantitative variables	11	Explain how quantitative variables were handled in the analyses. If applicable, describe which groupings were chosen and why	9-10
Statistical methods	12	(*a*) Describe all statistical methods, including those used to control for confounding	11
		(*b*) Describe any methods used to examine subgroups and interactions	11
		(*c*) Explain how missing data were addressed	N/A
		(*d*) If applicable, describe analytical methods taking account of sampling strategy	N/A
		(*e*) Describe any sensitivity analyses	N/A
**Results**			

For peer review only

Participants	13*	(a) Report numbers of individuals at each stage of study—eg numbers potentially eligible, examined for eligibility, confirmed eligible, included in the study, completing follow-up, and analysed	7
		(b) Give reasons for non-participation at each stage	7
		(c) Consider use of a flow diagram	N/A
Descriptive data	14*	(a) Give characteristics of study participants (eg demographic, clinical, social) and information on exposures and potential confounders	12
		(b) Indicate number of participants with missing data for each variable of interest	N/A
Outcome data	15*	Report numbers of outcome events or summary measures	
Main results	16	(*a*) Give unadjusted estimates and, if applicable, confounder-adjusted estimates and their precision (eg, 95% confidence interval). Make clear which confounders were adjusted for and why they were included	12
		(*b*) Report category boundaries when continuous variables were categorized	9
		(*c*) If relevant, consider translating estimates of relative risk into absolute risk for a meaningful time period	N/A
Other analyses	17	Report other analyses done—eg analyses of subgroups and interactions, and sensitivity analyses	11
**Discussion**			
Key results	18	Summarise key results with reference to study objectives	12-14
Limitations	19	Discuss limitations of the study, taking into account sources of potential bias or imprecision. Discuss both direction and magnitude of any potential bias	17-18
Interpretation	20	Give a cautious overall interpretation of results considering objectives, limitations, multiplicity of analyses, results from similar studies, and other relevant evidence	15-18
Generalisability	21	Discuss the generalisability (external validity) of the study results	17
**Other information**			
Funding	22	Give the source of funding and the role of the funders for the present study and, if applicable, for the original study on which the present article is based	20

*Give information separately for cases and controls in case-control studies and, if applicable, for exposed and unexposed groups in cohort and cross-sectional studies.

Note: An Explanation and Elaboration article discusses each checklist item and gives methodological background and published examples of transparent reporting. The STROBE checklist is best used in conjunction with this article (freely available on the Web sites of PLoS Medicine at http://www.plosmedicine.org/, Annals of Internal Medicine at http://www.annals.org/, and Epidemiology at http://www.epidem.com/). Information on the STROBE Initiative is available at www.strobe-statement.org.

For peer review only

BMJ Open

Prevalence and determinants of hyperglycemia among adults in Bangladesh: results from a population-based national survey

Journal:	*BMJ Open*
Manuscript ID	bmjopen-2019-029674.R2
Article Type:	Research
Date Submitted by the Author:	29-Jun-2019
Complete List of Authors:	Islam, Jessica; World Health Organization Country Office for Bangladesh, ; University of North Carolina at Chapel Hill Gillings School of Global Public Health, Department of Epidemiology<br>Zaman, MM; World Health Organization, Dhaka, Bangladesh,<br>Bhuiyan, Mahfuz; World Health Organization Country Office for Bangladesh<br>Haq, Syed; Bangabandhu Sheikh Mujib Medical University, Rheumatology<br>Ahmed, Shamim; Bangabandhu Sheikh Mujib Medical University<br>Al-Qadir, Zahid; Bangabandhu Sheikh Mujib Medical University
**Primary Subject Heading**:	Epidemiology
Secondary Subject Heading:	Diabetes and endocrinology, Global health
Keywords:	non-communicable diseases, chronic disease, raised blood glucose, Bangladesh

SCHOLARONE™
Manuscripts

Prevalence and determinants of hyperglycemia among adults in Bangladesh: results from a population-based national survey

Jessica Y. Islam[1,2], M. Mostafa Zaman*[2], Mahfuzur Rahman Bhuiyan[2], Syed Atiqul Haq[3], Shamim Ahmed[3], Zahid- Al- Quadir[3]

[1]Department of Epidemiology, UNC Gillings School of Global Public Health, Chapel Hill, NC; Email: islamjy@email.unc.edu

[2]World Health Organization, Dhaka, Bangladesh; Email: zamanm@who.int; Email: mahfuzdoc@gmail.com

[3]Department of Rheumatology, Bangabandhu Sheikh Mujib Medical University, Shahbag, Dhaka, Bangladesh. Email: haqsyedatiqul@gmail.com; Email: dr_shamim_ahmed@yahoo.com; Email: drzahid1981@yahoo.com

Corresponding Author:
Jessica Yasmine Islam, MPH
The University of North Carolina at Chapel Hill
Gillings School of Global Public Health
Department of Epidemiology
Chapel Hill, NC, USA 27599-7400
Email: islamjy@email.unc.edu

Manuscript word count: 4,200

For peer review only

For peer review only - http://bmjopen.bmj.com/site/about/guidelines.xhtml

Abstract

Objectives: With the increasing burden of non-communicable diseases in low- and middle-income countries, biological risk factors such as hyperglycemia are a major public health concern in Bangladesh. Hyperglycemia is an excess of glucose in the bloodstream and is often associated with type-2 diabetes mellitus. Nationally representative data of hyperglycemia prevalence starting from age ≥18 years are currently unavailable for Bangladeshi adults. The objective of this study was to assess the prevalence and determinants of hyperglycemia among adults in Bangladesh aged ≥18 years.

Study Design: Cross-sectional, population-based study

Setting and Participants: Data for this analysis were collected in November-December of 2015, from a population-based nationally representative sample of 1843 adults, aged ≥18 years, from both urban and rural areas of Bangladesh. Demographic information, capillary blood glucose, blood pressure, height, weight, waist circumference, and treatment history were recorded.

Primary Outcome Measures: Hyperglycemia was defined as a random capillary blood glucose level of ≥11.1mmol/L (i.e. in the diabetic range) or currently taking medication to control type-2 diabetes, based on self-report.

Results: Overall, the prevalence of hyperglycemia was 5.5% (95% CI: 4.5-6.6) and was significantly higher among urban (9.8%, 95% CI: 7.7-12.2) than rural residents (2.8%, 95% CI: 1.9-3.9). The age-standardized prevalence of hyperglycemia was 5.6% (95% CI: 4.6-6.8). Among both urban and rural residents, the associated determinants of hyperglycemia included hypertension and abdominal obesity. About 5% of the total population self-reported to have

For peer review only - http://bmjopen.bmj.com/site/about/guidelines.xhtml

been previously diagnosed with type-2 diabetes; among these adults, over 25% were not taking medications to control their diabetes.

Conclusions: Our study found that about 1 out of 20 Bangladeshi adults aged ≥18 years have hyperglycemia. To control and prevent the development of type-2 diabetes, data from this study can be used to inform public health programming and provide descriptive information on surveillance of progress towards controlling diabetes in Bangladesh.

Keywords: non-communicable diseases, Bangladesh, hyperglycemia, diabetes, chronic disease

For peer review only

Article Summary

Strengths and Limitations of the Study

- This study utilized a multistage, geographically clustered, probability-based sampling approach to produce nationally representative data for Bangladesh.

- Currently, nationally representative data for the prevalence of hyperglycemia in the diabetic range is unavailable for adults aged 18-29 years. A strength of our study is that we included Bangladeshi adults aged 18 years and above to obtain novel data on the prevalence of hyperglycemia, and relevant non-communicable disease risk factors

- We were able to estimate the prevalence of hyperglycemia using capillary blood glucose measured at random. However, we were unable to measure the prevalence of prediabetes and diabetes as we did not obtain blood sugar levels using standardized methods, such as fasting blood glucose or 2-hour post-prandial measurements.

- Due to the cross-sectional nature of the study design, we were unable to assess temporality of risk factors identified and our outcomes of interest.

- We assessed type-2 diabetes medication history based on self-report and we were able to obtain medicine strips or vials records or prescription records of participants to confirm the self-reported data.

For peer review only - http://bmjopen.bmj.com/site/about/guidelines.xhtml

Background

Globally, diabetes mellitus (DM), characterized by hyperglycemia, is a leading cause of premature mortality and disability. Globally, almost half of all deaths attributable to high blood glucose occur before the age of 70 years, and the WHO estimates that diabetes was the seventh leading cause of mortality in 2016. The global prevalence of DM has been on the rise over the past several decades[1 2]. Estimates reflect the global prevalence of diabetes has nearly doubled among adults aged 18 years and above, rising from 4.7% in 1980 to 8.5% in 2014[3]. This growing burden is most prominent in low- and middle-income countries particularly the Indian sub-continent [4], which accounts for close to one-fifth of all diabetes cases worldwide. The prevalence of diabetes in this region is projected to increase by 71% by 2035[5]. In Bangladesh, specifically, the International Diabetes Federation projects the prevalence of diabetes will increase to more than 50% in the next 15 years[5].

The increasing prevalence of diabetes among Bangladeshi adults over the past few decades has been documented: Based on a meta-analysis of studies conducted from 1995 – 2010, the prevalence of diabetes among Bangladeshi adults aged 30 years and above increased from 4% in 1995 to 2000 and 5% in 2001 to 2005 to 9% in 2006 to 2010[6]. Studies to assess the burden of diabetes have been conducted in Bangladesh in both urban and rural populations over the last decades[7]. However, national data on the prevalence of diabetes or hyperglycemia in the diabetic range starting at age 18 years are currently unavailable[7-10]. These data are valuable for monitoring progress made towards one of the nine global non-communicable disease (NCD) targets of the year 2025, set forth by the World Health Organization's (WHO) NCD Global Monitoring Framework: To observe a 0% increase in age-standardized prevalence of hyperglycemia or diabetes among persons aged ≥18 years[11]. As such in response to the WHO Global Action Plan for the Prevention and Control of NCDs[12], descriptive epidemiological data on the burden of hyperglycemia among adults starting at 18 years are needed to monitor the national progress of interventions implemented to reduce the

For peer review only

burden of DM in Bangladesh[13-15]. Here, using a nationally representative sample, we present data on prevalence and determinants of hyperglycemia by various sociodemographic factors such as age, sex, and area of residence among Bangladeshi adults aged 18 years and above, residing in both urban and rural areas of the country. Additionally, we explore treatment patterns and control of hyperglycemia among participants in our sample of Bangladeshi adults.

Methods

Data for this analysis were collected as part of a national assessment of the burden of musculoskeletal disorders in Bangladesh conducted by investigators from the Bangabandhu Sheikh Mujib Medical University (BSMMU) with technical assistance from the WHO Country Office for Bangladesh, as previously described[16]. The study was a population-based cross-sectional study carried out from November to December 2015 and followed the WHO STEP-wise approach to Surveillance of NCD risk factors (STEPS)[17]. The target population of this survey was men and women aged ≥18 years residing in rural and urban areas of Bangladesh. The exclusion criteria included tourists and the institutionalized, including residents of hospitals, prisons, nursing homes, and army barracks.

<u>Sampling Methods</u>

To obtain a population-based sample of Bangladesh, this survey adopted a multistage, geographically clustered, probability-based sampling approach. Population statistics were obtained using the updated national census conducted by the Bangladesh Bureau of Statistics (BBS) in 2009 [18]. To obtain our primary sampling unit (PSU), we utilized the following geographical distribution described: In Bangladesh, there are seven divisions, which are the largest administrative units of the country. Each division is divided into several districts (*Zila*) and within each district, there are several sub-districts (*Upazila*). Within sub-districts, *mauzas* and *mahallas* (commonly known as neighborhoods or blocks) are the smallest units within

For peer review only - http://bmjopen.bmj.com/site/about/guidelines.xhtml

defined territories in rural and urban areas respectively. *Mauzas* and *mahallas* were considered the PSU for the study's sampling approach. The households within the *mauzas* and *mahallas* were the secondary sampling units. We utilized the BBS' definition of a household which is as follows: "a dwelling in which persons either related or unrelated were living together and taking food from the same kitchen"[18].

Sample size estimation

The power analysis and sample size calculations were completed based on the standardized approach outlined in the WHO STEPS methodology [17]. Using the WHO STEPS methodology, the minimum number of participants required was 296 in each group (rural males, rural females, urban males, and urban females). Assuming a design effect of 1.5 adjusted within cluster population homogeneity, the necessary sample size was 1776. We assumed a response rate of 90% and determined we would need to contact at least 1973 adults. For simplicity, our target sample size was 2000. Twenty PSUs (8 urban and 12 rural) were randomly selected from 7 divisions of the country, with the probability proportional to the population size of each division. In each PSU, 100 consecutive households were selected. The even numbered households were designated as a "male household" and odd numbered households as a "female household." Finally, one male or female was approached to participate from each respective household as designated.

Data collection

Through a structured survey, we collected data on the following topics: musculoskeletal disorders[19], health history, and demographic data such as age, area of residence, education, current (last 12 months) occupation, tobacco use, and physical activity. Physical measurements such as height, weight, waist circumference, blood glucose levels, and blood pressure were collected. To measure blood glucose levels, we obtained random blood glucose samples [3], per

For peer review only - http://bmjopen.bmj.com/site/about/guidelines.xhtml

the clinical guidelines of diabetes diagnostic criteria of Bangladesh[20]. Capillary blood samples were consistently taken from the index finger of the right arm using a glucometer, namely Accu-chek Advantage (Roche Diagnostics Division, Grenzacherstrasse, Switzerland).

Each participant's history of diabetes was assessed based on self-report. Specifically, participants were asked: 1. Have you ever been diagnosed with diabetes by a health care professional? 2. If yes, are you receiving treatment for diabetes? Treatment history of diabetes was confirmed by prescription, including medicine strips or insulin injection vials, or medical records. The questionnaire was translated from English to Bengali, adapted and validated per standard procedure. Data collection procedures were standardized across study sites through coordinated training of field staff conducted by epidemiologists, study physicians, and WHO staff members.

Blood pressure was measured by a trained field interviewer using the LifeSource UA-767+ blood pressure monitor, as recommended by the WHO, and appropriately sized arm cuffs. Blood pressure measurements were consistently taken on each participant's right arm at the level of the heart and elbow-assisted, while the participant was in a seated position. The initial measurement was performed after five minutes of rest. After two minutes, the second measurement was taken. The mean of these two blood pressure readings was utilized as the final blood pressure for each participant.

Following data collection, participants scheduled a visit with the study research physician within the following five days. The research physician assessed the participant's medical history, either through self-report or using medical records when possible. Study physicians examined each participant to confirm the results of data collection through classical symptom assessment. When necessary, the participant was also evaluated by the divisional investigator for a second opinion of relevant diagnoses.

Patient and public involvement

For peer review only - http://bmjopen.bmj.com/site/about/guidelines.xhtml

There was no patient or public involvement in the implementation of this study or interpretation of analytic results.

Outcome definitions

Our primary outcome of interest was the prevalence of hyperglycemia. We utilized the American Diabetes Association (ADA) guidelines to define a diagnosis of hyperglycemia using random or casual plasma glucose test and symptom review by the study physician [21]. An individual was considered to have hyperglycemia if the plasma glucose level was 11.1 mmol/L or higher (i.e. in the diabetic range) and/or if they self-reported to take diabetes medication. Our secondary outcome of interest was the prevalence of self-reported type-2 diabetes mellitus. An individual was categorized as diabetic if they self-reported to have been previously diagnosed with diabetes by a health care provider.

Covariates

The following variables were assessed as covariates for analysis: area of residence, sex, age, education, occupation, wealth index, body mass index (BMI), BP, and waist circumference. Education was categorized into four groups: no education, primary education (completed grade ≤5), secondary education (completed ≤ grade 10), and above secondary education (completed ≥ grade 12). Each participant's occupation was categorized into five groups, including: professional employment (field staff, police officer, guard, doctor, engineer, professional, businessman, desk job), unemployed or retired, industrial worker or day laborer, housewife, and other (shop keeper, weaver, driver, student, beggar, cook, carpenter, tailor, migrant workers and fishermen).

Data on physical activity was collected based on self-report. First, respondents were asked the number of days they engaged in vigorous, moderate, or light physical activity throughout a typical week. The following definitions were used to define (1) vigorous, (2)

For peer review only - http://bmjopen.bmj.com/site/about/guidelines.xhtml

moderate, and (3) light physical activity, respectively: (1) vigorous activity was defined as any activity that caused a large increase in breathing or heart rate, if continued for at least 10 minutes (e.g. running, carrying heavy loads, digging or construction work); (2) moderate activity was defined as any activity that caused a small increase in breathing or heart rate, if continued for at least 10 minutes (brisk walking or carrying light loads); and (3) light physical activity was defined as activities such as office work. Next, we asked participants to estimate how many minutes per day they engaged in the activity. Metabolic equivalent of task (MET)-minute was calculated using the STEPS protocol[22] as follows: one minute of light activity was equivalent to 1 MET-minute; one minute in moderate-intensity activities was equivalent to 4 MET-minutes, and one minute of vigorous-intensity was equivalent to 8 MET-minutes. Physical activity was then categorized based on total MET-minutes per week. Participants who spent 3000 or more MET-minutes per week were categorized in the vigorous physical activity group, 600-3000 MET-minutes were categorized as moderate physical activity, and <600 MET-minutes were categorized as low physical activity.

The wealth index was constructed using principal component analysis. Asset information collected covered information on household ownership of nineteen items, including electricity, flush toilet, land telephone, cell phone, television, radio, refrigerator, car, motorcycle, washing machine, bicycle, sewing machine, wardrobe, table, bed or cot, chair or bench, watch or clock. Additionally, we assessed the main type of material used to build each participant's home (i.e. cement, tin, bamboo, or thatched straw). Each asset was assigned a weight (factor score) generated through principal components analysis, and the resulting asset scores were standardized to a normal distribution with a mean of zero and standard deviation of one. Each household was then assigned a score for each asset, and the scores were summed up; individuals were ranked according to the total score of the household in which they resided. The sample was then divided into quartiles from quartile one (lowest) to quartile four (highest).

For peer review only - http://bmjopen.bmj.com/site/about/guidelines.xhtml

Using height (centimeters) and weight (kilograms) measurements, we calculated BMI (height/weight^2). BMI was categorized in the following groups: underweight (≤ 18.5), normal (≤25), overweight (25.1-30) and obese (>30). Waist circumference was measured in centimeters (cm). Participants were categorized as abdominally obese if waist circumference was 90 cm and above for men, or 80 cm and above for women. Prehypertension was defined as SBP ≥120 mmHg but < 140 mmHg and/or DBP ≥ 80 mmHg but <90 mmHg and not taking antihypertensive medication at the time of the survey. We utilized the WHO's guidelines for cut-off points to define hypertension[23]. An individual was considered to have hypertension if systolic blood pressure (SBP) was ≥140 mmHg (millimeters of mercury) and/or, diastolic blood pressure (DBP) ≥90 mmHg, and/or taking antihypertensive medication based on self-report.

Age-Standardized Prevalence Estimates

To facilitate comparison of overall hyperglycemia among Bangladeshi adults across global populations with different age compositions we calculated age-standardized prevalence estimates with 95% confidence intervals (CIs) using the WHO's World Standard Population [24]. The World Standards database (WHO 2000-2025) provided population estimates for 18 and 19 age groups, as well as single year ages. To derive single ages from the 5-year age group proportions publically available, we used the Beers "Ordinary" Formula[25]. The following formula was utilized for standardization:

$\sum pi*w_i / \sum w_i$, where p = observed prevalence and w = world population weight.

Data Analysis

Sociodemographic variables were presented with mean and standard deviation (SD) for continuous variables, and using proportions for categorical variables. For bivariate analyses, study participants were divided by sex and into four age groups (18-29, 30-44, 45-54 and ≥55

For peer review only - http://bmjopen.bmj.com/site/about/guidelines.xhtml

years). We calculated the prevalence of our primary outcome by key demographic variables and calculated 95% CIs using the binomial exact method.

To estimate determinants of hyperglycemia in the diabetic range, we computed prevalence ratios with Poisson regression using robust estimation of standard errors[26-28]. Potential variables for inclusion in the model were assessed using prior published literature and bivariate Poisson regression analysis; an arbitrary p-value of <0.10 was used as criteria to include the variable in the multivariable Poisson regression model to control for confounding effects. For multivariable Poisson regression models, adjusted prevalence ratios (aPR), and 95% CIs for each independent variable were calculated. Additionally, *<0.05* was used as the level of significance. Multivariable Poisson regression models were generated separately for urban and rural participants to account for possible effect measure modification. Collinearity was assessed using the variance inflation factor to ensure a strong linear relationship among independent variables included in the model was not present. All statistical procedures were performed using Stata/SE 15.0 (StataCorp LP, Texas, USA) software package.

Results

<u>Background Characteristics</u>

Of the 2000 adults approached, 1843 agreed to participate in our study leading to a response rate of 92.1%. Twenty-four female participants were pregnant and were dropped from subsequent analyses to ensure those with gestational diabetes were not included. Additionally, no participants reported having been previously diagnosed with type-1 diabetes. There were 892 (49.0%) male and 927 (50.9%) female respondents (Table 1). The age of our participants ranged from 18 to 90 years. The mean age and education level of participants were 40.5 (SD = 14.7) years and 5.7 (SD = 5.1) years, respectively. The majority of the study population was married (88.0%) and employed as either an industrial worker/day laborer (26.5%) or housewife (39.9%). Almost half of participants never used some form of tobacco. The majority of

For peer review only - http://bmjopen.bmj.com/site/about/guidelines.xhtml

participants engaged in vigorous physical activity over an average week (69.3%). The mean BMI was 22.1 (SD = 4.1) and the mean waist circumference was 78.4 cm (SD = 11.6). Overall, the mean systolic blood pressure was 116.1 mmHg (SD = 17.1) and diastolic blood pressure was 76.1 mmHg (SD = 10.5). The mean blood glucose level was 6.4 mmol/L (SD = 2.4). Figure 1 presents the distribution of blood glucose levels by various demographic factors.

Prevalence and Risk Factors for Hyperglycemia

The prevalence of hyperglycemia was 5.5% (95% CI: 4.5-6.6). This prevalence was significantly higher among urban participants (9.8%, 95%CI: 7.7-12.2) than rural participants (2.8%, 95% CI: 1.9-3.9) (Table 2) and increased as age increased (Figure 2). The highest prevalence of hyperglycemia was observed among those aged ≥55 years, at 8.2% (95% CI: 4.6-13.1) among men and 9.4% (95% CI: 5.4-14.8) among women. The age-standardized prevalence of hyperglycemia was 5.6% (95% CI: 4.6-6.8). The age-standardized prevalence of hyperglycemia among urban and rural residents was 10.5% (95% CI: 9.2-12.1) and 2.8% (95% CI: 2.1-3.7), respectively. Among men and women, the age-standardized prevalence of hyperglycemia was 4.9% (95% CI: 3.9-6.0) and 6.0% (95% CI: 4.9-7.2), respectively.

Self-Reported Diabetes Mellitus and Hyperglycemia

Ninety-five participants (5.2%) self-reported to have been previously diagnosed with type-2 diabetes by a health care provider. However, 25 of those participants did not meet our criteria of diagnosis of hyperglycemia as they did not take medication to control their diabetes and their plasma glucose was below 11.1 mmol/L. Therefore, 69.3% of those with hyperglycemia, per the study definition, were previously diagnosed with diabetes by a health care provider.

The proportion of men and women who were previously diagnosed with diabetes by a health care provider based on self-report was higher among urban residents (men: 8.4%;

For peer review only - http://bmjopen.bmj.com/site/about/guidelines.xhtml

women: 9,1%) than among rural residents (men: 2.9%; women: 3.0%) (Figure 3a). However, overall, the large majority (81.8%) reported they did not know if they had been previously diagnosed with diabetes; this proportion was higher among rural residents (87.8%) than urban residents (72.2%).

Among participants previously diagnosed with type-2 diabetes based on self-report, 72.6% reported taking medication to control their diabetes (Table 1). Urban women more frequently (96.7%) self-reported to take diabetes medication than urban men (62.1%) (Figure 3b). We were able to confirm 100% of participant's self-reported diabetes treatment history by checking prescriptions or medicine strips/vials.

Among participants who were categorized as hyperglycemic during study measurement, over one-third (37.9%) of urban men self-reported to have diabetes, however, they did not take any medication to control their diabetes. Among rural participants, the proportion of women who did not take medication to control their self-reported diabetes was higher (52.9%) than men (31.3%). Although three-quarters of self-reported diabetic participants reported taking medication to control their diabetes, 31% continued to have high blood sugar levels indicating uncontrolled diabetes at study measurement (Figure 4).

Determinants of Hyperglycemia

Table 2 presents the results of multivariable Poisson regression with robust variance analyses to identify determinants of hyperglycemia. Among urban participants, those of older age, lowest wealth quartile, hypertension, low physical activity, and with abdominal obesity based on waist circumference, were more likely to have hyperglycemia. The prevalence of hyperglycemia was significantly highest among those aged ≥55 years (aPR 3.92, 95% CI: 1.48-10.39) compared to individuals aged 18-29 years of age. When compared to those in the 4th (highest) wealth quartile, urban residents in the first (lowest) wealth quartile had 3.18 times the prevalence of hyperglycemia. For urban individuals with hypertension, the prevalence of

For peer review only - http://bmjopen.bmj.com/site/about/guidelines.xhtml

hyperglycemia was 2.65 (95% CI:1.30-5.38) times that of individuals without hypertension. The prevalence of hyperglycemia among those with low physical activity was 3.01 (95% CI: 1.42-6.38) times that of urban participants with vigorous physical activity. Abdominal obesity also significantly increased the prevalence of hyperglycemia among urban participants (aPR: 2.54, 95% CI: 1.35-4.77). Among rural participants, the only observed determinants of hyperglycemia were hypertension (aPR: 5.39, 95% CI: 1.94-14.96) and abdominal obesity (aPR: 2.95, 95% CI: 1.32-6.58).

Discussion

Using data from this nationally representative sample, we estimate that about one in twenty Bangladeshi adults aged ≥18 years have hyperglycemia. The prevalence of hyperglycemia was higher among urban residents (9.8%) than rural residents (2.8%). Determinants of hyperglycemia included older age, urban residence, abdominal obesity, low physical activity, and hypertension. As diabetes is characterized by hyperglycemia, targeting high-risk groups identified in this analysis could be prioritized for effective diabetes preventive programs in Bangladesh.

Bangladesh has adopted the goals and targets set forth by the WHO's Global Monitoring Framework for the Prevention and Control of NCDs for the year 2025[11]. One of these targets is to ensure there is a 0% increase in the age-standardized prevalence of hyperglycemia among adults aged ≥18 years by the year 2025. To our knowledge, the present study is the first to report national estimates on the prevalence of hyperglycemia starting at age 18 years in both urban and rural areas of Bangladesh. Data gathered from this national-level study are critical towards the measurement of progress towards the WHO's nine global NCD control targets for Bangladesh for 2025.

Globally, the number of adults living with diabetes has risen from 108 million in 1980 to 422 million in 2013, and low- and middle-income countries (LMICs) have seen the most rapid

For peer review only - http://bmjopen.bmj.com/site/about/guidelines.xhtml

For peer review only

rise in diabetes prevalence[3]. Several lifestyle factors have been attributed to the increase in prevalence across LMICs including, globalization of food production, extensive marketing of low-cost and energy-dense foods, increased sedentary behavior, and rapid urbanization[29]. In recent decades, the increase in the prevalence of diabetes in South Asia has been greater than that seen in high-income countries[4]. The prevalence of diabetes in adults across countries in South Asia is similar, excluding Nepal which has a low prevalence in comparison to neighboring countries (8.8% in India, 8.6% in Sri Lanka, 6.9% in Bangladesh, 7.9% in Bhutan, 6.9% in Pakistan, and 4.0% in Nepal)[30]. In our study, we observed a prevalence of 5.6% hyperglycemia in the diabetic range among adults aged 18 years and above. This prevalence is lower than previous studies conducted in Bangladesh and neighboring countries. In fact, a scoping review estimated the pooled prevalence of type-2 diabetes to be 7.4%[7]. Our estimated prevalence was lower due to a younger study population (18 years and above compared to the WHO estimate among adults aged 30 years and above), and a higher percentage of participants from rural areas (61.1%, as is representative of Bangladesh). Indeed, when we restrict our analytic sample to 30 years and above, the prevalence of hyperglycemia is 6.7%, which is similar to the 2017 WHO estimate of diabetes (6.9%). Significant heterogeneity in diabetes and its determinants may exist within Bangladesh due to variations in the level of urbanization by region, and socioeconomic status of specific sub-populations[31].

Important determinants of hyperglycemia in both urban and rural areas of our assessment included hypertension, low physical activity, and abdominal obesity. Interestingly, there was no association of diabetes identified for increasing BMI. This indicates that abdominal obesity may be a more significant factor to consider than BMI. Prior studies conducted in Bangladesh have also identified a positive association of central (or abdominal) obesity with diabetes[32]. Furthermore, our assessment found a decrease in prevalence of diabetes with increasing wealth quartile. Prior studies have conflicting findings on the risk of diabetes and other NCDs, among the wealthy based on demographic features such as the area of residence.

For peer review only - http://bmjopen.bmj.com/site/about/guidelines.xhtml

One prior study conducted in Bangladesh found that people from the highest wealth quintile were more likely to have diabetes than people from the lowest wealth quintile[33]. However, another found a high burden of selected NCDs, including diabetes, among the lowest wealth quintile populations in rural areas and wealthy populations in urban areas[34]. Further study is warranted to assess the reliability of wealth indices as a measurement of socioeconomic status and wealth among Bangladeshi adults.

In our study, a high proportion (~70%) of those with hyperglycemia self-reported to have been previously diagnosed with diabetes and therefore, aware of their condition. Additionally, 72% reported taking medication to control their diabetes. However, we found that almost one-third of those who self-reported to take medication for their diabetes continued to have hyperglycemia. Efforts should be made to ensure diabetics in Bangladesh are treated for their condition and secondary prevention of complications of diabetes, such as diabetic retinopathy. This is of particular concern in developing countries where resources are limited and cost-effective solutions for chronic disease treatment should be prioritized. A recently published study found that healthcare expenditure in persons with diabetes in Bangladesh is six times higher than in persons without diabetes [14]. Prevention and management of diabetes are likely to be a cost-saving approach for Bangladesh through the utilization of community health workers adequately trained to effectively screen for, and identify, people with diabetes [35].

This study has several strengths. Data collected for our study was of a nationally representative sample indicating our results are generalizable to the population of Bangladeshi adults aged 18 years and above. Additionally, due to our large sample size, we were able to conduct subgroup analyses to identify urban and rural differences. However, several limitations should also be considered when interpreting the results of this analysis. We were unable to measure the prevalence of prediabetes and diabetes directly as we did not obtain blood sugar levels using standardized methods, such as fasting blood glucose or 2-hour post-prandial measurements. Further, we did not assess each participant’s history of classical symptoms of

For peer review only - http://bmjopen.bmj.com/site/about/guidelines.xhtml

hyperglycemia, which is necessary to diagnose diabetes according to the ADA guidelines[21]. We were unable to measure known determinants of type-2 diabetes factors such as diet or family history of diabetes. Future studies should consider the addition of glycosylated hemoglobin measurement when assessing the prevalence of diabetes as this method could provide a more long-term and stable diagnosis of diabetes mellitus. Finally, due to the cross-sectional nature of this study, we were unable to define temporality of certain determinants of hyperglycemia identified and therefore, unable to assess causality.

Conclusion

Data from this nationally-representative sample of Bangladeshi adults aged 18 years and above will be critical to informing the progress of NCD control in Bangladesh per the WHO's Global Monitoring Framework and goals for 2025. As our data were collected in late 2015, more recent studies to estimate the prevalence of diabetes mellitus or hyperglycemia are warranted. Recent changes in risk factor distribution coupled with aging of the population may have led to changes prevalence of diabetes mellitus not reflected in our results, which will be important in measuring our progress as we approach 2025. We found that about one in twenty Bangladeshi adults aged ≥18 years have hyperglycemia. Among urban residents, we found that about one in ten Bangladeshi adults aged ≥18 years have hyperglycemia. Bangladeshi adults with hypertension and abdominal obesity are high-risk groups for the development of diabetes and should be targeted for routine screening for diabetes. Preventive methods such as lifestyle changes and medication should be recommended by primary care providers in Bangladesh to avoid the future development of CVDs among this group. In order to control the prevalence of hyperglycemia in the diabetic range, and reduce the burden of diabetes or associated risk factors, national initiatives such as training community health workers to deliver primary care and implementing universal health coverage should be implemented to curb the spread of NCDs in Bangladesh.

For peer review only - http://bmjopen.bmj.com/site/about/guidelines.xhtml

For peer review only

For peer review only - http://bmjopen.bmj.com/site/about/guidelines.xhtml

Figures

Figure 1: Urban and Rural differences in the distribution of blood glucose levels based on random capillary blood measurement among (A) All Participants, and (B) Men and Women (n = 1819)

Figure 2: Prevalence of hyperglycemia among Bangladeshi adults aged 18 years and above by sex and age group, 2015 (n = 1819)

Figure 3: Bangladeshi adults aged 18 years and above with (A) self-reported diabetes and (B) self-reported diabetics on diabetes medications, 2015

Figure 4: Self-reported diabetics aged 18 years or older who take diabetes medication with hyperglycemia on study measurement (≥11.0mmol/L)

For peer review only

For peer review only - http://bmjopen.bmj.com/site/about/guidelines.xhtml

Abbreviations

WHO: World Health Organization

ADA: American Diabetes Association

aPR: Adjusted prevalence ratio

BBS: Bangladesh Bureau of Statistics

BMI: Body mass index

BP: Blood pressure

BSMMU: Bangabandhu Sheikh Mujib Medical University

CI: Confidence interval

CVD: Cardiovascular disease

MET: Metabolic equivalent

mmHg: Millimeter of mercury

mmol/L: Millimoles per liter

NCD: Non-communicable disease

SBP: Systolic blood pressure

DBP: Diastolic blood pressure

PSU: Primary sampling unit

SD: Standard deviation

STEPS: STEPwise approach to Surveillance

For peer review only

Footnotes

Ethics approval and consent to participate: Ethical guidelines as outlined by the Declaration of Helsinki were followed throughout the study. Ethical clearance was obtained from the Institutional Review Board of Bangabandhu Sheikh Mujib Medical University (BSMMU) (Protocol Number: 1100). We obtained permission from the relevant administrative units of the surveyed districts. Orientations with community leaders (elected representatives of the local government offices) were conducted prior to data collection for community engagement in the study's implementation process. Written (or thumb impression if unable to write) consent was obtained from the respondents in Bangla as per BSMMU Institutional Review Board (IRB) guidelines.

Availability of data and materials: The de-identified participant data used and/or analyzed during the current study are available from the corresponding author on reasonable request. Please contact M. Mostafa Zaman at zamanm@who.int for further information and guidelines.

Competing Interests: The authors declare no competing interests. The authors alone are responsible for views expressed in this article and they do not necessarily represent the views, decisions or policies of the institutions with which they are affiliated.

Funding: The study was conducted with the technical and financial assistance of the World Health Organization Country Office for Bangladesh.

Authors contributions: JYI: conceptualized the manuscript, analyzed data, interpreted results critically, and drafted the manuscript. MMZ: designed the study, interpreted results critically, guided manuscript writing, and critically reviewed it. MRB, SAH, SA, ZAQ: trained the field team, implemented the survey, processed and analyzed data, and reviewed the manuscript.

Acknowledgments: The authors thank Mr. Hassanuzzaman Khan for his efforts on data management.

For peer review only

References

1. Disease GBD, Injury I, Prevalence C. Global, regional, and national incidence, prevalence, and years lived with disability for 354 diseases and injuries for 195 countries and territories, 1990-2017: a systematic analysis for the Global Burden of Disease Study 2017. *Lancet (London, England)* 2018;392(10159):1789-858. doi: 10.1016/S0140-6736(18)32279-7

2. Ogurtsova K, da Rocha Fernandes JD, Huang Y, et al. IDF Diabetes Atlas: Global estimates for the prevalence of diabetes for 2015 and 2040. *Diabetes research and clinical practice* 2017;128:40-50. doi: 10.1016/j.diabres.2017.03.024

3. Global report on diabetes. Geneva, Switzerland: World Health Organization 2016.

4. Jayawardena R, Ranasinghe P, Byrne NM, et al. Prevalence and trends of the diabetes epidemic in South Asia: a systematic review and meta-analysis. *BMC public health* 2012;12:380. doi: 10.1186/1471-2458-12-380

5. IDF diabetes atlas sixth edition Brussels, Belgium Interational Diabetes Federation, 2013.

6. Saquib N, Saquib J, Ahmed T, et al. Cardiovascular diseases and type 2 diabetes in Bangladesh: a systematic review and meta-analysis of studies between 1995 and 2010. *BMC public health* 2012;12:434. doi: 10.1186/1471-2458-12-434

7. Biswas T, Islam A, Rawal LB, et al. Increasing prevalence of diabetes in Bangladesh: a scoping review. *Public health* 2016;138:4-11. doi: 10.1016/j.puhe.2016.03.025

8. Rahman MS, Akter S, Abe SK, et al. Awareness, treatment, and control of diabetes in Bangladesh: a nationwide population-based study. *PloS one* 2015;10(2):e0118365. doi: 10.1371/journal.pone.0118365

9. Akter S, Rahman MM, Abe SK, et al. Prevalence of diabetes and prediabetes and their risk factors among Bangladeshi adults: a nationwide survey. *Bulletin of the World Health Organization* 2014;92(3):204-13, 13A. doi: 10.2471/BLT.13.128371

For peer review only - http://bmjopen.bmj.com/site/about/guidelines.xhtml

10. Zaman MM, Choudhury SR, Ahmed J, et al. Blood glucose and cholesterol levels in adult population of Bangladesh: Results from STEPS 2006 survey. *Indian heart journal* 2016;68(1):52-6. doi: 10.1016/j.ihj.2015.06.031

11. NCD Global Monitoring Framework: World Health Organization; 2017 [Available from: http://www.who.int/nmh/global_monitoring_framework/en/ accessed 09/29 2017.

12. Global action plan for the prevention and control of noncommunicable diseases 2013-2020. Geneva, Switzerland: World Health Organization, 2013.

13. Siddique MKB, Islam SMS, Banik PC, et al. Diabetes knowledge and utilization of healthcare services among patients with type 2 diabetes mellitus in Dhaka, Bangladesh. *BMC health services research* 2017;17(1):586. doi: 10.1186/s12913-017-2542-3

14. Shariful Islam SM, Lechner A, Ferrari U, et al. Healthcare use and expenditure for diabetes in Bangladesh. *BMJ Glob Health* 2017;2(1):e000033. doi: 10.1136/bmjgh-2016-000033

15. Haghparast-Bidgoli H, Shaha SK, Kuddus A, et al. Protocol of economic evaluation and equity impact analysis of mHealth and community groups for prevention and control of diabetes in rural Bangladesh in a three-arm cluster randomised controlled trial. *BMJ open* 2018;8(8):e022035. doi: 10.1136/bmjopen-2018-022035

16. Islam JY, Zaman MM, Haq SA, et al. Epidemiology of hypertension among Bangladeshi adults using the 2017 ACC/AHA Hypertension Clinical Practice Guidelines and Joint National Committee 7 Guidelines. *Journal of human hypertension* 2018;32(10):668-80. doi: 10.1038/s41371-018-0087-5

17. Chronic Disease and Health Promotion: STEP wise approach to surveillance (STEPS). Geneva: World Health Organization.

18. Population and Housing Census 2001, updated in 2009. Dhaka, Bangladesh: Bangladesh Bureau of Statistics 2009.

For peer review only

For peer review only - http://bmjopen.bmj.com/site/about/guidelines.xhtml

19. Goycochea-Robles MV, Sanin LH, Moreno-Montoya J, et al. Validity of the COPCORD core questionnaire as a classification tool for rheumatic diseases. *J Rheumatol Suppl* 2011;86:31-5. doi: 10.3899/jrheum.100955

20. Guidelines for Care of Type 2 Diabetes Mellitus in Bangaldesh. Dhaka, Bangladesh: BIRDEM Clinical Research Group, 2003.

21. American Diabetes A. 2. Classification and Diagnosis of Diabetes. *Diabetes care* 2017;40(Suppl 1):S11-S24. doi: 10.2337/dc17-S005

22. Moniruzzaman M, Mostafa Zaman M, Islam MS, et al. Physical activity levels in Bangladeshi adults: results from STEPS survey 2010. *Public health* 2016;137:131-8. doi: 10.1016/j.puhe.2016.02.028

23. A global brief on Hypertension: Silent Killer, global public health crisis. Geneva, Switzerland: World Health Organization, 2013.

24. Ahmad OB, Boschi-Pinto C, Lopez AD, et al. Age Standardization of Rates: A New WHO Standard. GPE Discussion Paper Series: No31 Geneva, Switzerland: World Health Organization, 2001.

25. World (WHO 2000-2025) Standard Rockville, MD: National Cancer Institute: Surveillance, Epidemiology, and End Results Program; 2017 [Available from: https://seer.cancer.gov/stdpopulations/world.who.html accessed December 17 2017.

26. Coutinho LM, Scazufca M, Menezes PR. Methods for estimating prevalence ratios in cross-sectional studies. *Revista de saude publica* 2008;42(6):992-8.

27. Behrens T, Taeger D, Wellmann J, et al. Different methods to calculate effect estimates in cross-sectional studies. A comparison between prevalence odds ratio and prevalence ratio. *Methods Inf Med* 2004;43(5):505-9.

28. Barros AJ, Hirakata VN. Alternatives for logistic regression in cross-sectional studies: an empirical comparison of models that directly estimate the prevalence ratio. *BMC medical research methodology* 2003;3:21. doi: 10.1186/1471-2288-3-21

For peer review only

For peer review only - http://bmjopen.bmj.com/site/about/guidelines.xhtml

29. Singh PN, Arthur KN, Orlich MJ, et al. Global epidemiology of obesity, vegetarian dietary patterns, and noncommunicable disease in Asian Indians. *The American journal of clinical nutrition* 2014;100 Suppl 1:359S-64S. doi: 10.3945/ajcn.113.071571

30. Hills AP, Arena R, Khunti K, et al. Epidemiology and determinants of type 2 diabetes in south Asia. *Lancet Diabetes Endocrinol* 2018;6(12):966-78. doi: 10.1016/S2213-8587(18)30204-3

31. Chan JC, Malik V, Jia W, et al. Diabetes in Asia: epidemiology, risk factors, and pathophysiology. *Jama* 2009;301(20):2129-40. doi: 10.1001/jama.2009.726

32. Alam DS, Talukder SH, Chowdhury MA, et al. Overweight and abdominal obesity as determinants of undiagnosed diabetes and pre-diabetes in Bangladesh. *BMC Obes* 2016;3:19. doi: 10.1186/s40608-016-0099-z

33. Tareque MI, Koshio A, Tiedt AD, et al. Are the rates of hypertension and diabetes higher in people from lower socioeconomic status in Bangladesh? Results from a nationally representative survey. *PloS one* 2015;10(5):e0127954. doi: 10.1371/journal.pone.0127954

34. Biswas T, Islam MS, Linton N, et al. Socio-Economic Inequality of Chronic Non-Communicable Diseases in Bangladesh. *PloS one* 2016;11(11):e0167140. doi: 10.1371/journal.pone.0167140

35. Gaziano TA, Abrahams-Gessel S, Denman CA, et al. An assessment of community health workers' ability to screen for cardiovascular disease risk with a simple, non-invasive risk assessment instrument in Bangladesh, Guatemala, Mexico, and South Africa: an observational study. *The Lancet Global health* 2015;3(9):e556-63. doi: 10.1016/S2214-109X(15)00143-6

For peer review only - http://bmjopen.bmj.com/site/about/guidelines.xhtml

Table 1: Background Characteristics of Bangladeshi adult participants, 2015 (n = 1819)

	Total (n = 1819)			Urban (n = 708)			Rural (n = 1111)		
Characteristic	Mean (SD)	n	%	Mean (SD)	n	%	Mean (SD)	n	%
Sex									
Male		892	49.0		345	48.7		547	49.2
Female		927	50.9		363	51.3		564	50.8
Age(years)	40.5 (14.7)			39.1 (13.9)			41.4 (15.1)		
Education (Years) [a]	5 (0 - 9)			8 (3-12)			4 (0-8)		
Marital Status									
Never Married		110	6.1		54	7.6		56	4.9
Married		1601	88.0		619	87.4		982	88.4
Separated/Divorced/Widowed		108	5.9		35	4.9		73	6.5
Occupation									
Professional employment [b]		279	15.2		189	26.7		90	8.1
Unemployed/retired		98	5.3		43	6.1		55	4.9
Industrial worker/Day Laborer		483	26.6		120	16.9		363	32.7
Housewife		726	39.9		247	34.9		479	43.2
Other [c]		232	12.8		109	15.4		123	11.1
Wealth Index [d]									
1st Wealth Quartile		407	22.4		110	15.5		297	26.7
2nd Wealth Quartile		533	29.3		171	24.2		362	32.6
3rd Wealth Quartile		429	23.6		179	25.3		250	22.5
4th Wealth Quartile		450	24.7		248	35.0		202	18.2
Tobacco Use [e]									
Never		859	47.2		389	54.9		470	42.3
Current Use		821	45.1		268	37.9		553	49.8
Past Use		139	7.6		51	7.2		88	7.9
Smoking Tobacco Use [f]									
Every day / Occasionally		494	27.2		169	23.9		325	29.3
Past Use		104	5.7		38	5.4		66	5.9
Never		1221	67.1		501	70.8		720	64.8
Smokeless Tobacco Use [g]									
Every day / Occasionally		529	29.1		150	21.2		379	34.1
Past Use		51	2.8		21	2.9		30	2.7
Never		1239	68.1		537	75.9		702	63.2
Physical Activity [h]									
Vigorous		1268	69.7		445	62.9		823	74.1
Moderate		464	25.5		234	33.1		230	20.7
Low		87	4.7		29	4.1		58	5.2

For peer review only - http://bmjopen.bmj.com/site/about/guidelines.xhtml

Body Mass Index [i]	22.1 (4.1)		23.3 (4.5)		21.3 (3.7)	
Waist Circumference (cm)	78.4 (11.6)		81.8 (12.6)		76.2 (10.3)	
Blood Pressure						
Systolic Blood Pressure (mmHg)	116.1 (17.1)		117.9 (16.6)		115.0 (17.3)	
Diastolic Blood Pressure (mmHg)	76.1 (10.5)		77.9 (10.9)		74.9 (10.1)	
Blood Glucose Level (mmol/l)	6.4 (2.4)		6.6 (2.9)		6.3 (2.1)	
Self-reported diabetes medication history [j]	69	72.6	50	80.6	19	57.6

Abbreviations: SD, standard deviation

[a] Calculated median and interquartile range for education as the data are skewed

[b] Professional occupation includes: Field staff, police officer, guard, doctor, engineer, professional, business man, desk job

[c] Other occupation includes: Shop keeper, weavers, driver, student, beggar, cook, carpenter, tailor, migrant workers and fishermen

[d] Wealth index was calculated using principal component analysis using data collected on household ownership of the following items: electricity, flushable toilet, land phone, cell phone, television, radio, refrigerator, private car, motor cycle, washing machine, bicycle, sewing machine, almirah/wardrobe, table, bed, chair/bench, watch/clock, as well as, type of main material used to build their homes roof, walls and floor

[e] Includes both smokeless tobacco and smoke tobacco

[f] Smoking tobacco use includes cigarettes, biri, hookah, etc.

[g] Smokeless tobacco use includes jarda, sada pata, pan mashala with tobacco leaf, gul etc.

[h] Measured in MET-minutes; 1 MET stands for the amount of oxygen you consume and the number of calories you burn at rest.

[i] Body mass index (BMI) calculated by weight in kilogram divided by height in meter squared

[j] Percentage reported out of participants who self-reported to have diabetes (Total n = 95; Urban n = 62; Rural n = 33)

For peer review only

Table 2: Determinants of hyperglycemia among Bangladeshi adults, 2015 (n = 1819)

	Total (n = 1819)		Urban (n = 708)		Rural (n = 1111)	
	Diabetes [a] Prevalence %	Adjusted PR [b] (95% CI)	Diabetes [a] Prevalence %	Adjusted PR [b] (95% CI)	Diabetes [a] Prevalence %	Adjusted PR [b] (95% CI)
**Characteristic**						
Area						
Urban	9.8	Ref.	-	-	-	-
Rural	2.8	0.44 (0.28 – 0.68)	-	-	-	-
Sex						
Male	4.9	Ref.	7.5	Ref.	3.3	Ref.
Female	5.9	1.05 (0.71 – 1.54)	11.9	1.26 (0.80 – 1.99)	2.2	0.61 (0.29 – 1.28)
Age (years)						
18 - 29	2.5	Ref.	2.5	Ref.	2.5	Ref.
30 - 44	4.9	1.48 (0.78 – 2.79)	8.4	2.55 (1.01 – 6.41)	2.5	0.76 (0.30 – 1.93)
45 - 54	7.4	2.18 (1.10– 4.31)	15.5	4.38 (1.62 – 11.59)	2.8	0.67 (0.25 – 1.83)
≥ 55	8.8	1.92 (0.95 – 3.86)	19.3	3.92 (1.48 – 10.39)	3.4	0.53 (0.17 – 1.67)
Educational Status						
No Education	3.4	Ref.	7.0	Ref.	2.1	Ref.
Primary Education	3.7	1.00 (0.53 – 1.86)	7.6	1.00 (0.44 – 2.30)	2.1	0.84 (0.30 – 2.30)
Secondary Education	6.9	1.67 (0.95 – 2.93)	12.1	1.94 (0.95 – 3.97)	2.9	1.01 (0.40 – 2.54)
Above Secondary Education	9.8	1.48 (0.77 – 2.84)	10.4	1.54 (0.71 – 3.33)	8.1	2.24 (0.68 – 7.41)
Wealth Index [c]						
1st Wealth Quartile	7.7	2.58 (1.57 – 4.24)	18.9	3.18 (1.80 – 5.62)	3.6	1.37 (0.53 – 3.49)
2nd Wealth Quartile	4.3	1.23 (0.71 – 2.14)	9.4	1.50 (0.82 – 2.77)	1.9	0.70 (0.24 – 2.05)
3rd Wealth Quartile	3.7	0.86 (0.47 – 1.58)	6.0	0.96 (0.48 – 1.92)	2.0	0.55 (0.18 – 1.73)
4th Wealth Quartile	6.6	Ref.	8.8	Ref.	3.9	Ref.
Blood Pressure						
Normal Blood Pressure	1.9	Ref.	3.5	Ref.	1.1	Ref.
Pre-Hypertension [d]	5.7	1.74 (1.00 – 3.01)	8.3	1.37 (0.69 – 2.74)	3.8	2.32(0.91 – 5.92)
Hypertension [e]	18.9	3.57 (2.01 – 6.34)	27.4	2.65 (1.30 – 5.38)	8.8	5.39(1.94 –14.96)
Physical Activity						
Vigorous	3.9	Ref.	6.9	Ref.	2.3	Ref.
Moderate	8.2	1.18 (0.78 – 1.77)	13.8	1.22 (0.77 – 1.93)	2.1	0.65 (0.22 – 1.88)
Low	14.9	3.04 (1.69 – 5.47)	20.7	3.01 (1.42 – 6.38)	12.1	2.58 (0.95 – 7.04)

For peer review only - http://bmjopen.bmj.com/site/about/guidelines.xhtml

Body Mass Index [f]						
Underweight (<18.5)	1.1	0.37 (0.12 – 1.13)	1.8	0.38 (0.07 – 1.95)	0.7	0.42 (0.09 – 2.02)
Normal (18.5 - 25)	4.7	Ref.	8.2	Ref.	2.8	Ref.
Overweight (25.1 - 30)	10.4	1.06 (0.68 – 1.65)	14.0	1.05 (0.61 – 1.80)	6.2	1.22 (0.51 – 2.91)
Obese (>30)	20.9	1.49 (0.87 – 2.57)	26.5	1.46 (0.81 – 2.64)	5.6	0.95 (0.12 – 7.60)
Waist Circumference (cm)						
Normal [g]	2.3	Ref.	3.8	Ref.	1.6	Ref.
Abdominally Obese [h]	13.3	2.49 (1.53 – 4.07)	18.1	2.54 (1.35 – 4.77)	6.8	2.95 (1.32 – 6.58)

Abbreviations: PR = prevalence ratio, CI = confidence intervals, Ref = referent category

[a] Hyperglycemia was defined as a capillary blood glucose level greater than or equal to 11.1 mmol/L or self-reported diabetes medication use

[b] Model adjusted for all variables included in table: sex, age, education, wealth index, blood pressure, body mass index, self-reported physical activity, and waist circumference

[c] Wealth index was calculated using principal component analysis using data collected on household ownership of the following items: electricity, flushable toilet, land phone, cell phone, television, radio, refrigerator, private car, motor cycle, washing machine, bicycle, sewing machine, almirah/wardrobe, table, bed, chair/bench, watch/clock, as well as, type of main material used to build their homes roof, walls and floor

[d] Pre-Hypertension was defined as SBP ≥120 mmHg but < 140 mmHg and/or DBP ≥ 80 mmHg but <90 mmHg and not taking anti-hypertensive medication at the time of the survey

[e] Hypertension was defined as systolic blood pressure (SBP) was ≥140 mmHg (millimeters of mercury) and/or, diastolic blood pressure (DBP) ≥90 mmHg and/or taking any-hypertensive medication

[f] Body mass index (BMI) calculated by weight in kilogram divided by height in meter squared

[g] Defined as <90 cm M; <80 cm F

[h] Defined as ≥90 cm M; ≥80 cm F

For peer review only

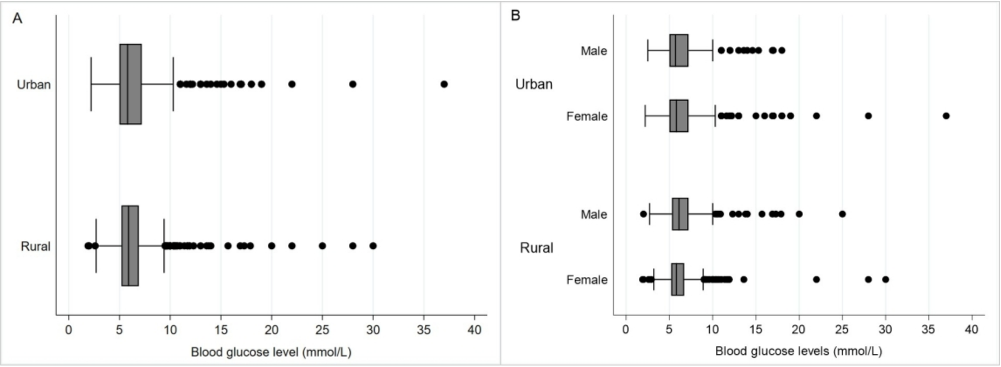

Figure 1: Urban and Rural differences in the distribution of blood glucose levels based on random capillary blood measurement among (A) All Participants, and (B) Men and Women (n = 1819)

327x119mm (150 x 150 DPI)

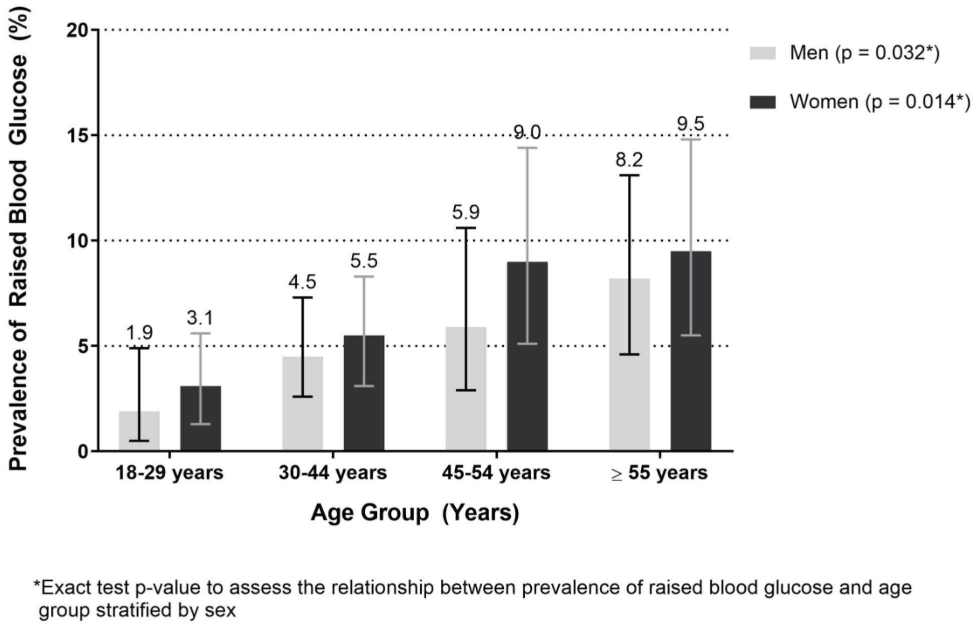

Figure 2: Prevalence of hyperglycemia among Bangladeshi adults aged 18 years and above by sex and age group, 2015 (n = 1819)

211x137mm (300 x 300 DPI)

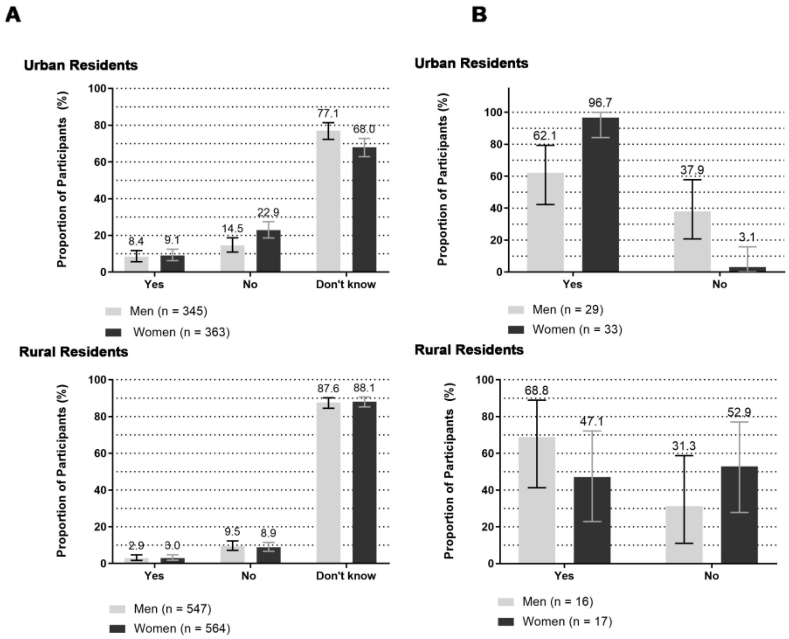

Figure 3: Bangladeshi adults aged 18 years and above with (A) self-reported diabetes and (B) self-reported diabetics on diabetes medications, 2015

284x190mm (300 x 300 DPI)

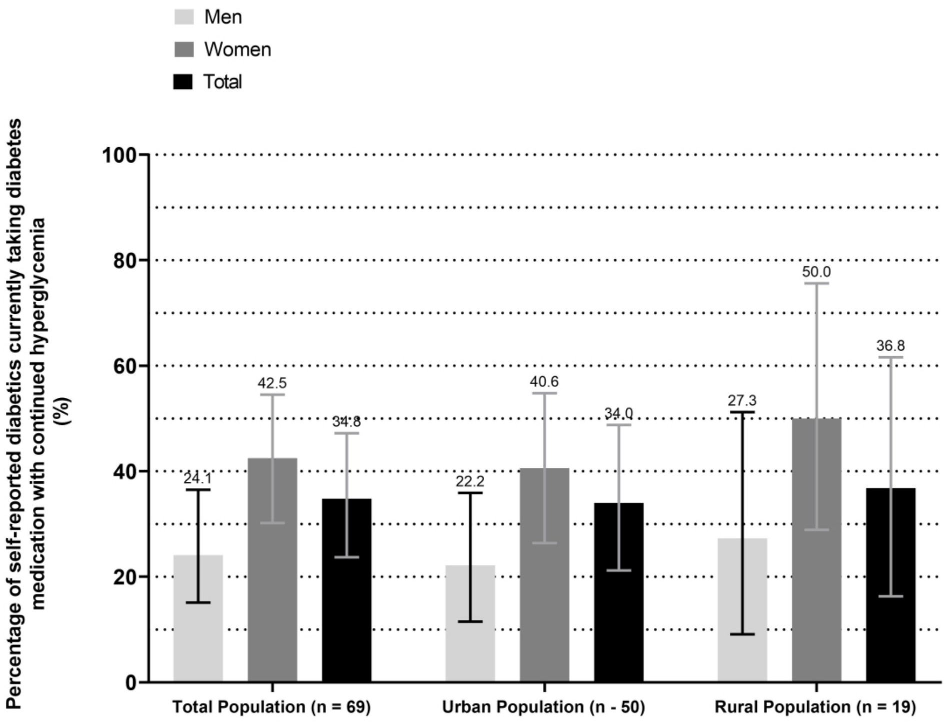

Figure 4: Self-reported diabetics aged 18 years or older who take diabetes medication with hyperglycemia on study measurement (≥11.0mmol/L)

284x210mm (300 x 300 DPI)

STROBE 2007 (v4) Statement—Checklist of items that should be included in reports of *cross-sectional studies*

Section/Topic	Item #	Recommendation	Reported on page #
**Title and abstract**	1	(*a*) Indicate the study's design with a commonly used term in the title or the abstract	2
		(*b*) Provide in the abstract an informative and balanced summary of what was done and what was found	2-3
**Introduction**			
Background/rationale	2	Explain the scientific background and rationale for the investigation being reported	5-6
Objectives	3	State specific objectives, including any prespecified hypotheses	6
**Methods**			
Study design	4	Present key elements of study design early in the paper	6
Setting	5	Describe the setting, locations, and relevant dates, including periods of recruitment, exposure, follow-up, and data collection	7
Participants	6	(*a*) Give the eligibility criteria, and the sources and methods of selection of participants	6
Variables	7	Clearly define all outcomes, exposures, predictors, potential confounders, and effect modifiers. Give diagnostic criteria, if applicable	8-9
Data sources/ measurement	8*	For each variable of interest, give sources of data and details of methods of assessment (measurement). Describe comparability of assessment methods if there is more than one group	7-10
Bias	9	Describe any efforts to address potential sources of bias	6-7 & 11
Study size	10	Explain how the study size was arrived at	7
Quantitative variables	11	Explain how quantitative variables were handled in the analyses. If applicable, describe which groupings were chosen and why	9-10
Statistical methods	12	(*a*) Describe all statistical methods, including those used to control for confounding	11
		(*b*) Describe any methods used to examine subgroups and interactions	11
		(*c*) Explain how missing data were addressed	N/A
		(*d*) If applicable, describe analytical methods taking account of sampling strategy	N/A
		(*e*) Describe any sensitivity analyses	N/A
**Results**			

For peer review only

Participants	13*	(a) Report numbers of individuals at each stage of study—eg numbers potentially eligible, examined for eligibility, confirmed eligible, included in the study, completing follow-up, and analysed	7
		(b) Give reasons for non-participation at each stage	7
		(c) Consider use of a flow diagram	N/A
Descriptive data	14*	(a) Give characteristics of study participants (eg demographic, clinical, social) and information on exposures and potential confounders	12
		(b) Indicate number of participants with missing data for each variable of interest	N/A
Outcome data	15*	Report numbers of outcome events or summary measures	
Main results	16	(*a*) Give unadjusted estimates and, if applicable, confounder-adjusted estimates and their precision (eg, 95% confidence interval). Make clear which confounders were adjusted for and why they were included	12
		(*b*) Report category boundaries when continuous variables were categorized	9
		(*c*) If relevant, consider translating estimates of relative risk into absolute risk for a meaningful time period	N/A
Other analyses	17	Report other analyses done—eg analyses of subgroups and interactions, and sensitivity analyses	11
**Discussion**			
Key results	18	Summarise key results with reference to study objectives	12-14
Limitations	19	Discuss limitations of the study, taking into account sources of potential bias or imprecision. Discuss both direction and magnitude of any potential bias	17-18
Interpretation	20	Give a cautious overall interpretation of results considering objectives, limitations, multiplicity of analyses, results from similar studies, and other relevant evidence	15-18
Generalisability	21	Discuss the generalisability (external validity) of the study results	17
**Other information**			
Funding	22	Give the source of funding and the role of the funders for the present study and, if applicable, for the original study on which the present article is based	20

*Give information separately for cases and controls in case-control studies and, if applicable, for exposed and unexposed groups in cohort and cross-sectional studies.

Note: An Explanation and Elaboration article discusses each checklist item and gives methodological background and published examples of transparent reporting. The STROBE checklist is best used in conjunction with this article (freely available on the Web sites of PLoS Medicine at http://www.plosmedicine.org/, Annals of Internal Medicine at http://www.annals.org/, and Epidemiology at http://www.epidem.com/). Information on the STROBE Initiative is available at www.strobe-statement.org.

For peer review only
